# scMustree: a multi-scale functional hierarchy for single-cell transcriptomic analysis

**DOI:** 10.1093/bioinformatics/btag672

**Published:** 2026-09-10

**Authors:** Yunpei Xu, Shaokai Wang, Liqing Ding, Hong-Dong Li, Jianxin Wang

**Affiliations:** School of Computer Science and Engineering, Central South University, Changsha 410083, China; Xiangjiang Laboratory, Changsha 410205, China; School of Computer Science and Engineering, Central South University, Changsha 410083, China; Xiangjiang Laboratory, Changsha 410205, China; School of Computer Science and Engineering, Central South University, Changsha 410083, China; Xiangjiang Laboratory, Changsha 410205, China; School of Computer Science and Engineering, Central South University, Changsha 410083, China; Xiangjiang Laboratory, Changsha 410205, China; School of Computer Science and Engineering, Central South University, Changsha 410083, China; Xiangjiang Laboratory, Changsha 410205, China

## Abstract

**Motivation:**

Resolving cellular heterogeneity requires methods that capture both discrete cell types and their functional relationships. Existing single-cell clustering approaches often produce flat partitions, limiting their ability to reveal rare cell states and continuous biological transitions. Here, we introduce scMustree, a computational framework that constructs a multi-scale hierarchy of single-cell transcriptomes, enabling integrated analysis of cellular organization and functional relationships. scMustree employs a top-down iterative decomposition to isolate transcriptionally homogeneous populations, followed by a bottom-up merging strategy guided by cluster-specific functional rankings—derived from differential expression and an isolation-forest-based scoring mechanism that quantifies functional distinctness. This unified approach captures lineage structures, functional similarities, and transitional states.

**Results:**

Across 11 benchmark datasets, scMustree achieves competitive clustering accuracy while offering substantially enhanced biological interpretability. In diverse biological systems, it uncovers biologically consistent hierarchies and identifies previously uncharacterized cell states, including fibroblast and T-cell subsets in cancer and lipid-associated microglial populations in Alzheimer’s disease. By integrating structural and functional information, scMustree provides a scalable and biologically grounded framework for multi-resolution exploration of single-cell ecosystems, enabling discovery of rare and disease-relevant cell states across diverse biological contexts.

**Availability and implementation:**

Freely available at Github (https://github.com/xuyp-csu/scMustree) and Zenodo (https://zenodo.org/records/17480562).

## 1 Introduction

Single-cell RNA sequencing (scRNA-seq) has revolutionized our ability to resolve cellular heterogeneity within tissue microenvironments (Kinker *et al.* 2020). Clustering algorithms, as a cornerstone of scRNA-seq analysis, group cells by transcriptional similarity to enable critical downstream applications, including cell type annotation, differential expression analysis, and intercellular communication inference.

Current clustering approaches fall into two paradigms: Traditional frameworks (e.g. Seurat (Hao *et al.* 2024), Scanpy (Wolf *et al.* 2018)) remain widely adopted, employing highly variable gene selection with linear dimensionality reduction followed by graph-based community detection. In recent years, building upon these foundations, deep learning-enhanced methods have emerged to further improve clustering performance by capturing complex, nonlinear patterns in the data. For instance, DESC (Li *et al.* 2020) optimizes feature representation through autoencoders, scDCC (Tian *et al.* 2021) integrates Gaussian mixture models for joint feature learning and clustering, and scGNN (Wang *et al.* 2021) employs graph neural networks to model cellular topological relationships. These methods enhance clustering precision through nonlinear feature extraction.

Despite these advancements, three fundamental challenges persist in single-cell clustering analysis. First, algorithmic dependence on predefined cluster numbers contradicts the biological reality of unknown cell-type quantities (Yu *et al.* 2022), risking oversimplification of cellular heterogeneity and obscuring rare or transitional populations. Moreover, optimization-driven convergence criteria often fail to align with biologically meaningful distinctions, potentially yielding biologically irrelevant groupings. Second, current methods primarily produce discrete partitions, overlooking the continuous nature of cellular differentiation and functional transitions. As a result, important biological insights, such as functional similarities (Wang *et al.* 2022), lineage trajectories (Bibby *et al.* 2022), hierarchical structures (Mayer *et al.* 2023), or subtle gradations between cell states (Jerber *et al.* 2021), may remain hidden. This limitation underscores the need for approaches capable of capturing both discrete groupings and the underlying relational structure among cells. Third, evaluation metrics exhibit inherent biases due to resolution discrepancies in reference annotations (Luecken and Theis 2019): coarse-grained labels inadequately assess performance, while fine-grained annotations struggle with increasing data scales and class imbalance (Xu *et al.* 2023). This biological complexity highlights the critical need for multi-scale analytical frameworks that can integrate hierarchical and functional information to comprehensively interpret single-cell data.

Recent advancements in leveraging hierarchical structures for optimizing single-cell analysis have shown promise. For instance, Zhong *et al.* (2025) constructed cell type evolutionary trajectories—a “cell type tree of life”—by integrating cross-species single-cell RNA-seq data across broad phylogenetic distances, demonstrating that such hierarchical structures inherently reflect underlying biological organization. In addition, several methods have been developed to construct and analyze hierarchical cellular representations. AGAC (Zhou *et al.* 2025) employs a mixed attention mechanism to hierarchically aggregate graph structural features and multi-scale node attribute features, enabling adaptive fusion of information across network layers for improved clustering performance. scSHC (Grabski *et al.* 2023) improves hierarchical clustering through statistical significance analysis but is sensitive to the initial clustering configuration. Similarly, CHOIR (Sant *et al.* 2025) adopts a top-down hierarchical tree construction approach, integrating random forest classifiers with permutation testing to mitigate over-clustering. However, its initialization process may be influenced by initial clustering outcomes and may not fully capture relationships among clusters at the same hierarchical level. SEAT (Chen and Li 2023) establishes a hierarchy based on the cell graph and structural entropy reduction, yet faces fundamental limitations in quantifying intercellular relationships within high-dimensional sparse datasets. CHETAH (De Kanter *et al.* 2019) and MRtree (Peng *et al.* 2021) require pre-existing reference datasets or multi-resolution clustering results. While Wu and Wu (2020) proposed hierarchy-sensitive metrics (wRI/wNMI), their work focuses on evaluation rather than construction of hierarchical representations.

To address these challenges, we propose *scMustree* (*s*ingle-*c*ell *Mu*lti-*s*cale *tree*), a computational framework that constructs multi-scale hierarchies to infer and visualize the organizational structure of cellular populations. Rather than treating cell populations as isolated entities, scMustree organizes them into a multi-scale hierarchy, where each node or branch captures distinct functional characteristics or transitional potential. By analyzing the topology of this hierarchy—such as branch relationships, divergence points, and nested substructures—researchers can perform an in-depth exploration of functional associations, lineage progressions, and intermediate cellular states. Through comprehensive benchmarking across 11 single-cell datasets, scMustree achieves competitive clustering accuracy while offering substantially enhanced biological interpretability. Notably, it identifies previously unannotated cellular subtypes and states, such as lipid-associated microglial states in Alzheimer’s disease and activated muscle satellite cells within the tumor stroma. These discoveries are supported by differential expression analysis and cross-dataset correlation. Collectively, these results establish scMustree as a powerful tool for multi-scale single-cell analysis across diverse biological contexts.

## 2 Materials and methods

### 2.1 Overview of scMustree

**Figure 1:**
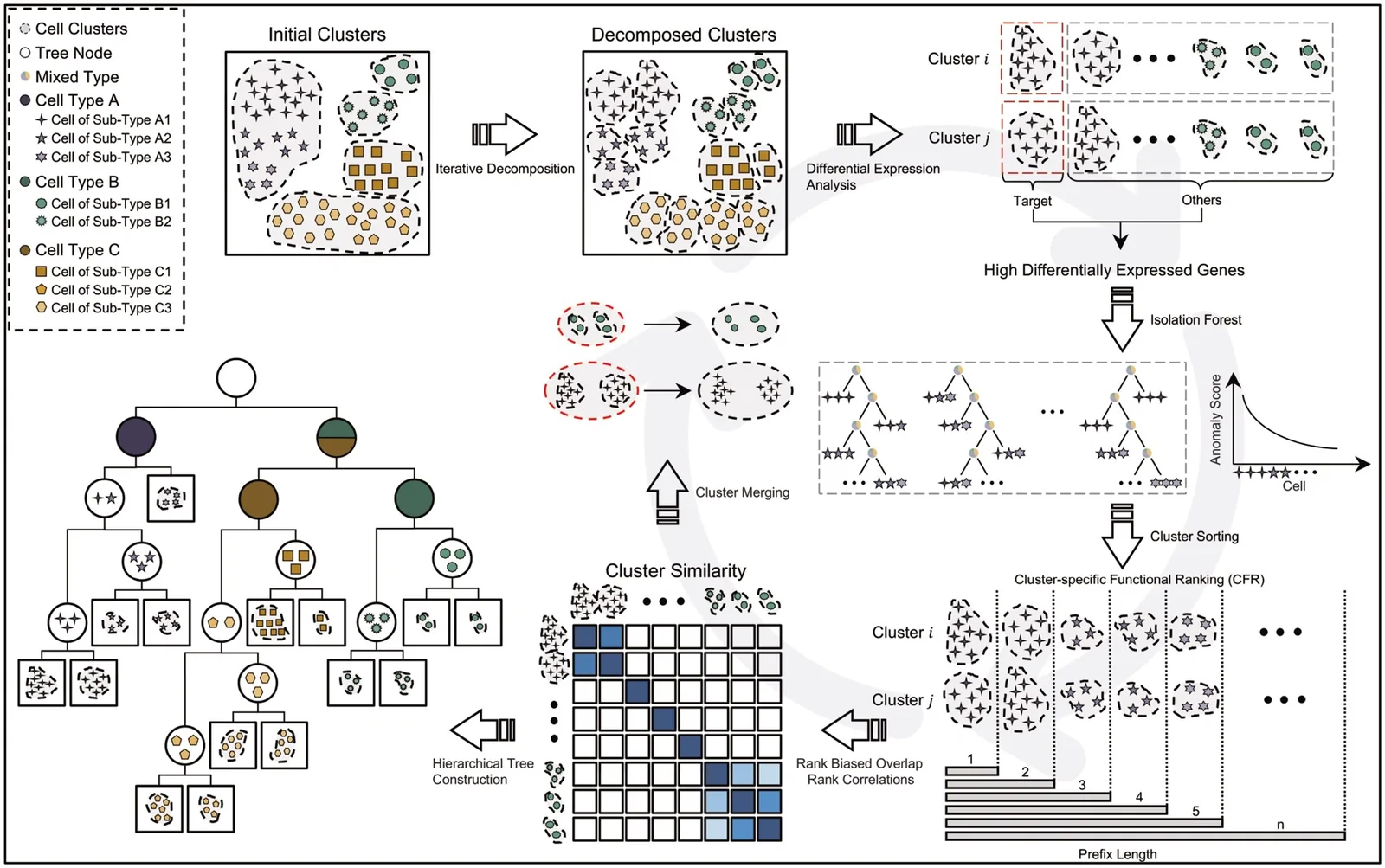
Overview of scMustree. The scMustree pipeline begins with top-down iterative clustering, decomposing initial clusters into reliable decomposed populations (leaf populations). Next, it identifies differentially expressed genes (DEGs) for each cluster, constructs decision trees based on their expression patterns, and defines the cluster-specific functional ranking (CFR). Using pairwise CFR rank correlations, scMustree performs bottom-up hierarchical merging, resulting in a multi-scale hierarchy that captures functional relationships between cell types.

In contrast to conventional approaches that analyze scRNA-seq data based on cell discrete partitions, scMustree introduces a new perspective that treats single-cell analysis as the exploration of hierarchical organization, capturing both global structures and fine-grained cellular relationships. This approach naturally reveals intercellular functional relationships through branching patterns, enabling multi-scale and multi-resolution analysis of cellular composition and providing deeper insights into cellular heterogeneity and functional diversity. A schematic of the scMustree pipeline can be found in Fig. 1. scMustree integrates three core components: (1) the determination of terminal populations through cluster decomposition, (2) the inference of cluster-specific functional orders (CFR) using decision trees, and (3) rank correlation-guided merging. The methodology for each step is described below.

#### 2.1.1 Step 1: data preprocessing

The pipeline begins with data preprocessing. The gene expression matrix is preprocessed by first filtering out lowly expressed genes that may not provide informative signals. Specifically, only genes detected in at least three cells are retained for downstream analysis. Next, each cell’s raw UMI counts are divided by its total library size to yield relative frequencies, and subsequently scaled by the median library size across all cells. The resulting normalized values are then log-transformed using the natural logarithm after adding a pseudo-count of 1. This step ensures that the data are suitable for downstream analysis.

#### 2.1.2 Step 2: terminal population determination via cluster decomposition

scMustree employs a top-down, iterative refinement strategy to generate reliable decomposed clusters (which form the terminal populations of the hierarchy). Starting from the preprocessed expression matrix *X* (of dimensions *N* cells ×  *G* genes), the method first partitions all cells into I-clusters (initial clusters). Each I-cluster is then iteratively decomposed in two rounds of clustering to produce D-clusters (decomposed clusters), which are designated as the terminal populations. This number of decomposition rounds was chosen based on an empirical analysis of cluster size reduction across multiple datasets. We observed that after two decompositions, the average cluster size typically falls below 1% of the total data (see Fig. S1, available as supplementary data at *Bioinformatics* online for the size distribution of decomposed clusters across all benchmark datasets).

To perform the cluster decomposition, scMustree repeatedly applies a fast single-cell clustering module. At each round, the module takes the cells in the current cluster as input and uses all retained genes after the initial filtering step. Principal component analysis (PCA) is then performed on this cell subset to obtain the top principal components that capture the major variance structure within the current cluster. Thus, although no additional round-specific gene selection is performed, the low-dimensional representation is dynamically updated at each decomposition step because PCA is recomputed within the current cell subset. In the resulting PCA-reduced space, an undirected cell graph is constructed using Euclidean distance and the k-nearest neighbors (KNN) algorithm. Finally, a graph-based community detection algorithm (e.g. Louvain) is employed to assign cluster labels to the cells.

#### 2.1.3 Step 3: cluster-specific functional gene selection

We posit that genes differentially expressed in a cluster relative to others reflect the functional programs active in those cells, and thus select them as cluster-specific functional genes. For each cluster $D_{i}$ in D-clusters, scMustree first identifies genes with expression rates greater than or equal to the median expression rate in $D_{i}$. Let $X_{D_{i}}^{g}=\{x_{1}^{g},x_{2}^{g},\ldots ,x_{N_{i}}^{g}\}$ represents the expression vector of gene *g* across $N_{i}$ cells in cluster $D_{i}$, where $x_{j}^{g}$ denotes the expression value of gene *g* in cell *j*. The number of expressing cells for gene *g* in $D_{i}$ is computed as: $P_{D_{i}}^{g}=\sum_{j=1}^{N_{i}}I(x_{j}^{g}>0)$, where I(·) is an indicator function that equals 1 if $x_{j}^{g}>0$ and 0 otherwise. Genes satisfying $P_{D_{i}}^{g}\geq \text{median}(\{P_{D_{i}}^{g^{\prime }}|g^{\prime }\in G\})$ are retained. For these genes, we compute intra-cluster ($\mu_{D_{i}}^{g}$) and extra-cluster ($\mu_{D_{i}^{\prime }}^{g}$) mean expression values ($D_{i}^{\prime }$ denotes all cells outside $D_{i}$). The log2-fold change is calculated as: $F_{D_{i}}^{g}={\;\text{log}\;}_{2}(\mu_{D_{i}}^{g}+1)-{\;\text{log}\;}_{2}(\mu_{D_{i}^{\prime }}^{g}+1)$. Genes are ranked by $F_{D_{i}}^{g}$, and the top $n_{\text{top}}=\text{min}(|\{g|F_{D_{i}}^{g}>0\}|,100)$ genes with positive fold changes are selected as cluster-specific functional genes ($G_{D_{i}}$).

#### 2.1.4 Step 4: functional ranking calculation using functional isolation score

To evaluate the relationship between each cluster and a reference cluster-specific functional gene set, scMustree constructs an isolation forest model for each functional gene set. Unlike its conventional application for anomaly detection, scMustree exploits the isolation mechanism to quantify how specifically cells represent a reference functional program (Liu *et al.* 2008). Specifically, for a given gene set $G_{D_{i}}$, an Isolation Forest model is trained using the expression matrix of all cells in the dataset restricted to the genes in $G_{D_{i}}$. The cells exhibiting this functional program tend to occupy a distinct region in the corresponding functional feature space. Consequently, these cells can be separated from the background population using shorter isolation paths, indicating stronger representation of the reference functional signature. Therefore, the isolation process in scMustree measures functional specificity relative to the reference gene signature. The functional isolation score for cell *j* under the reference functional gene set $G_{D_{i}}$ is calculated as:


(1) $$S_{j}^{G_{D_{i}}}=2^{\frac{E(h(j))}{c(N)}}$$

where h(j) is the path length (edge count from root to leaf node), E(h(j)) is the average path length across all trees, and c(N) represents the expected path length for *N* cells:


(2) $$\{\begin{array}{cc} 2H(N-1)-\frac{2(N-1)}{N}, & N>2\\ 1, & N=2\\ 0, & \text{otherwise} \end{array}$$

where H(N−1) is the harmonic number that can be estimated by ln(N−1)+0.5772156649 (Euler’s constant) (Liu *et al.* 2008). Under this definition, cells with shorter isolation paths generate smaller functional isolation scores, indicating stronger consistency with the reference functional program represented by $G_{D_{i}}$.

For each cluster $D_{j}$, scMustree summarizes the cell-level functional isolation scores by taking their median:


(3) $$S_{D_{j}}^{G_{D_{i}}}=\text{median}(\{S_{m}^{G_{D_{i}}}|m\in D_{j}\})$$

where a smaller value indicates that cluster $D_{j}$ contains cells that more strongly exhibit the functional expression pattern represented by $G_{D_{i}}$. Because all clusters are evaluated using the same Isolation Forest model for a given $G_{D_{i}}$, these median scores provide a comparable measure of functional similarity among clusters. Clusters are therefore sorted in ascending order of $S_{D_{j}}^{G_{D_{i}}}$, producing a cluster functional ranking (CFR), where populations at the top of the ranking represent the closest functional neighbors of the reference cluster $D_{i}$:


(4) $${\text{CFR}}_{D_{i}}=\text{sort}(\{S_{D_{1}}^{G_{D_{i}}},S_{D_{2}}^{G_{D_{i}}},\ldots ,S_{D_{k}}^{G_{D_{i}}}\})$$

where *k* is the total number of clusters in the D-clusters.

#### 2.1.5 Step 5: cluster similarity calculation using Rank-Biased Overlap (RBO)

Because the top-ranked clusters in each CFR represent the closest functional neighbors of the reference cluster, the agreement between the top portions of two CFRs reflects the similarity of their functional neighborhoods. scMustree therefore uses Rank-Biased Overlap (RBO), which emphasizes top-ranked agreement, to quantify functional similarity between clusters. Specifically, the similarity between clusters $D_{i}$ and $D_{j}$ is computed using their corresponding CFRs, ${\text{CFR}}_{D_{i}}$ and ${\text{CFR}}_{D_{j}}$, via the Rank-Biased Overlap (RBO) metric (Webber *et al.* 2010):


(5) $${\text{RBO}}_{i,j}=(1-p)\sum_{d=1}^{k}p^{\left(d-1\right)}\frac{\left|{\text{CFR}}_{D_{i}}^{\left(:d\right)}\cap {\text{CFR}}_{D_{j}}^{\left(:d\right)}\right|}{d}$$

where *d* is the ranking depth, *k* is the total number of nodes (i.e. the number of elements in ${\text{CFR}}_{D_{i}}$, and the parameter *p* (with 0<p<1) controls the rate at which the weight decays with increasing depth. A smaller *p* results in faster decay of contributions from increasing ranking depths and therefore places greater emphasis on top-ranked populations, whereas a larger *p* allows deeper ranking positions to contribute more substantially.

To determine the appropriate value of *p*, we aim to ensure that the closest functional neighbors at the top of the CFR contribute the majority of the RBO similarity. First, we quantify the contribution associated with each ranked element. This distinction is necessary because an element appearing at rank position *d* contributes not only to the overlap at depth *d*, but also to all subsequent prefix overlaps where this element remains included. Therefore, the contribution weight associated with the element located at rank position *d* is calculated by accumulating its influence across the current depth and all subsequent depths:


(6) $$W_{\text{RBO}}(d)=\frac{1-p}{p}\sum_{i=d}^{\infty }\frac{p^{i}}{i}$$

where the summation from *d* to infinity reflects that the element at rank position *d* contributes to every prefix overlap with depth greater than or equal to *d*. Subsequently, the cumulative contribution of the first *d* ranked elements is obtained by summing the contributions of the elements appearing in the top-*d* positions, which gives:


(7) $$W_{\text{RBO}}(1:d)=\sum_{j=1}^{d}W_{\text{RBO}}(j)=\frac{1-p}{p}\sum_{j=1}^{d}\sum_{i=j}^{\infty }\frac{p^{i}}{i}$$

which, after rearrangement, gives:


(8) $$W_{\text{RBO}}(1:d)=1-p^{d-1}+\frac{1-p}{p}\cdot d\cdot (\text{ln}\frac{1}{1-p}-\sum_{i=1}^{d-1}\frac{p^{i}}{i})$$

After counting the number of terminal populations dominated by different types across all datasets (Fig. S2, available as supplementary data at *Bioinformatics* online), we find that the median number of clusters corresponding to each type in most datasets is close to 5. Therefore, we focus on the top 5 clusters’ rankings as the primary parameter (accounting for 90%) for measuring local population-to-population functional relationships. That is, d=5 and the value of *p* satisfying $W_{\text{RBO}}(1:d)\geq 0.9$.

#### 2.1.6 Step 6: iterative merging for tree construction

scMustree iteratively executes Steps 3–4 for all D-clusters. Guided by the RBO similarity scores from Step 5, the algorithm merges the two most similar clusters (populations) into a new merged group that aggregates all constituent cells. The merged node’s cluster-specific functional gene set ($G_{D_{i}}$) is recalculated through Steps 3–4 to reflect its updated cellular composition. This recalculation-triggered similarity update propagates through Step 5, refreshing all pairwise RBO relationships across the remaining populations. The process iterates until convergence, where all populations coalesce into a single root group, yielding the complete multi-scale hierarchy.

## 3 Results

### 3.1 Benchmarking multi-scale hierarchical construction

To comprehensively assess the performance of multi-scale hierarchical construction methods, we conduct benchmarking through algorithmic accuracy and biological interpretability. For algorithmic comparisons, we select a range of representative methods, including the multi-resolution-based Louvain algorithm, dimensionality-reduction-based hierarchical clustering (scSHC), structure entropy optimization (SEAT), and deep learning-based approaches such as scDCC and scGNN. Our experiments utilize 11 single-cell datasets, covering diverse anatomical regions (e.g. airway, hippocampus) and species models (e.g. human, mouse). Notably, a subset of cell type annotations in these datasets is validated through experimental techniques such as immunofluorescence staining, flow cytometry sorting, and cross-platform dataset consistency analysis, ensuring high biological credibility. The specifics of these datasets can be found in Table S1, available as supplementary data at *Bioinformatics* online.

**Figure 2:**
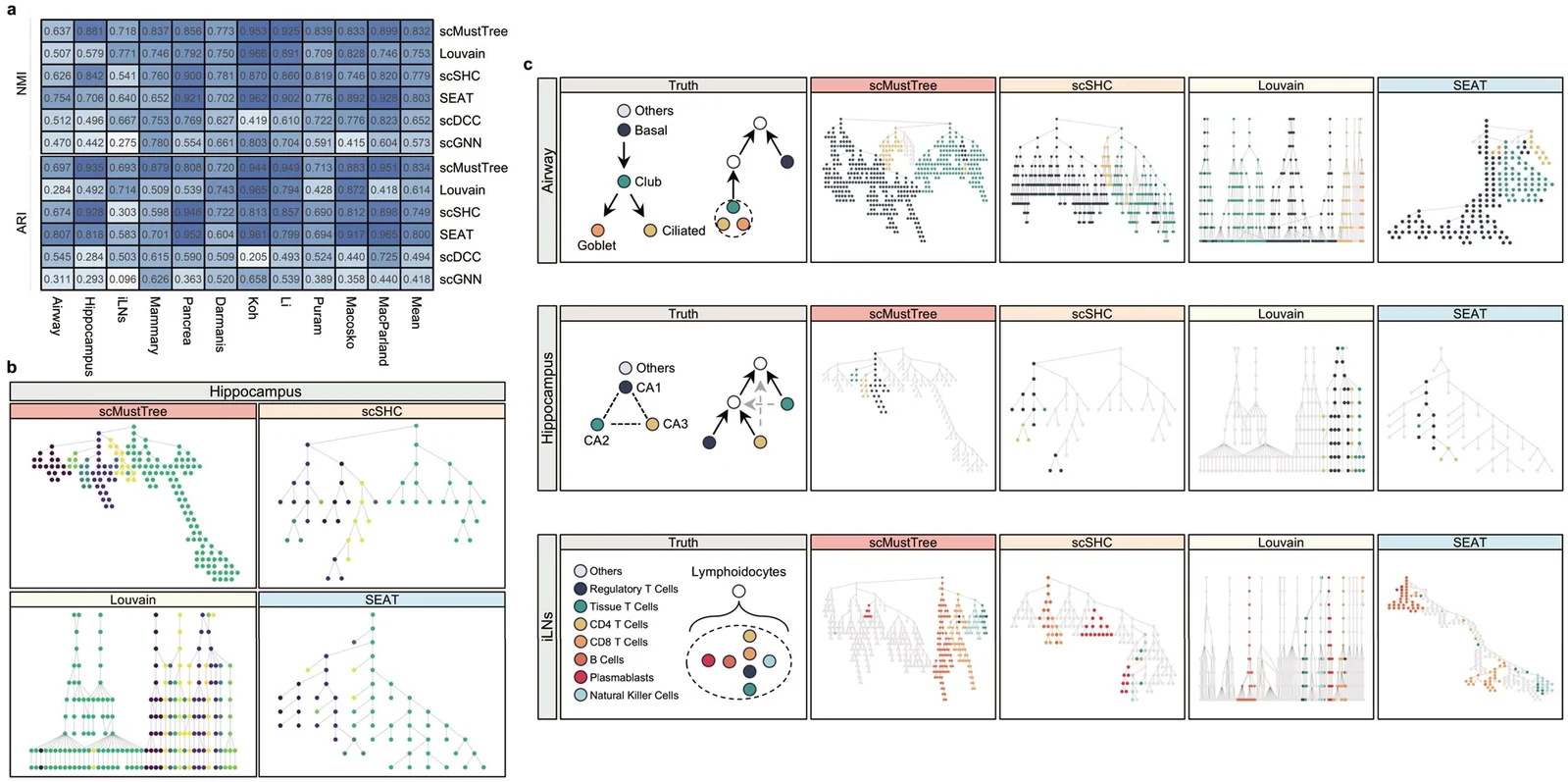
Benchmarking multi-scale hierarchical construction methods in algorithmic precision and biological interpretability. (a) Algorithmic precision comparison of four hierarchical methods methods (Louvain, scSHC, SEAT, scMustree) and two deep learning-based approaches (scDCC, scGNN) in terms of NMI and ARI. (b) Cell type-resolved hierarchies generated by these methods in the Hippocampus dataset. Nodes are colored by dominant cell types. (c) Biological interpretability assessment across three datasets with reliable ground-truth relationships (left panels) and corresponding method performance (right panels).

Algorithmic accuracy is quantified using normalized mutual information (NMI) and adjusted Rand index (ARI) against cell type annotations. For hierarchical methods, including scMustree, scSHC, and SEAT, we evaluate the hierarchy using an oracle pruning protocol designed to assess whether the inferred tree contains biologically meaningful partitions at an appropriate resolution. Specifically, we apply a bottom-up tree-pruning algorithm to extract a set of non-overlapping nodes whose cell assignments best match the reference cell-type annotations, and use the selected node set as the cluster assignment for computing ARI and NMI. This protocol, which balances node purity and global recall while discouraging over-fragmentation, is fully described in Supplementary Note 1. For the non-hierarchical method Louvain, which produces flat partitions but depends on the resolution parameter, we evaluate a range of resolution values and report the best-performing result. Implementation details and parameter settings for all comparative methods are given in Supplementary Note 2, available as supplementary data at *Bioinformatics* online. Benchmarking results are shown in Fig. 2a. scMustree achieves the best overall performance, with average NMI and ARI values of 0.832 and 0.834, and outperforms the second-ranked method (SEAT: NMI = 0.803, ARI = 0.800) by 3.61% and 4.25%, respectively. In addition, compared to partition-based clustering methods, hierarchical approaches show significant advantages in clustering accuracy. These methods can reflect the relationships between clusters, which helps mitigate the issues of over-clustering (incorrectly dividing cells from the same type into multiple clusters) and under-clustering (incorrectly merging cells from different types into a single cluster). For instance, the hierarchy of the Louvain algorithm is composed of clustering results at different resolutions. The algorithm may distinguish clusters that are hard to define clearly under high-resolution settings. At the same time, in low-resolution mode, it may merge clusters that are originally distinct but belong to the same type. The detailed performance of Louvain across a range of resolutions is provided in Tables S2 and S3, available as supplementary data at *Bioinformatics* online, which report NMI and ARI values, respectively, for each dataset and indicate the selected resolution. As a result, this algorithm demonstrates acceptable clustering performance across different datasets.

We visualize the hierarchies generated by the four hierarchical methods across all datasets, coloring nodes according to the cell types they primarily represent. Visualization results for the hippocampus dataset are shown in Fig. 2b, with visualizations for other datasets provided in Fig. S3, available as supplementary data at *Bioinformatics* online. scMustree achieves branch-level type discrimination, with each major branch predominantly containing a single cell type.

To evaluate the biological interpretability of constructed hierarchies, we analyze the topological consistency between hierarchical architectures and reliable cell-type relationships derived from original dataset studies and known biological relationships. We use three representative datasets with well-defined cellular relationships: (1) Developmental trajectories (Airway): lineage-committed transitions from basal to secretory cells; (2) Spatial subtypes (Hippocampus): Anatomically distinct neuronal subtypes (CA1–CA3); (3) Functional modules (iLNs): Lymphocyte subtypes with coordinated immune responses.

The airway dataset published by Montoro *et al.* is predominantly composed of basal cells and club cells (Montoro *et al.* 2018). To investigate differentiation dynamics, the authors developed an innovative “pulse-seq” methodology that integrates single-cell RNA sequencing (scRNA-seq) with in vivo genetic lineage tracing, enabling temporal monitoring of cell fate transitions. Their lineage tracing analysis revealed a hierarchical differentiation pathway: basal cells initially differentiate into club cells, which subsequently generate goblet and ciliated cells (Fig. 2c). Among the hierarchies generated by the four methods, the Louvain algorithm, operating through multi-resolution clustering, faces inherent constraints in lineage reconstruction. While lower clustering resolutions naturally yield fewer cell groups, this parameter reduction does not guarantee biologically meaningful merging of distinct cell types. While SEAT captures the overall differentiation hierarchy, it inaccurately positions goblet and ciliated cells of equivalent differentiation states at distant branches. scSHC exhibits structural similarities to scMustree in preserving differentiation relationships. However, scSHC incorrectly clusters other cell types primarily along the branch dominated by club cells. In the Hippocampus dataset (Habib *et al.* 2016), focusing on the CA1, CA2, and CA3 subregions that form interconnected neuronal circuits, scMustree successfully clusters these subregions into three distinct branches within a unified sub-hierarchy, aligning with their known biological interplay. In contrast, scSHC and Louvain disperse these subregions across unrelated branches, while SEAT clusters them within a single branch but fails to maintain CA1 spatial coherence. Analysis of the iLNs dataset (Huang *et al.* 2021), which contains diverse immune cell populations, specifically highlights scMustree’s precision in segregating lymphocyte types into discrete branches of a shared sub-hierarchy. Other methods exhibit erroneous adjacency relationships between distinct lymphocyte types (Fig. 2c).

In summary, scMustree demonstrates significant advantages in both algorithmic accuracy and biological interpretability. Its superior clustering performance and accurate representation of biological relationships between cell types establish scMustree as a powerful tool for constructing multi-scale cellular hierarchies in single-cell data analysis.

### 3.2 scMustree identifies multi-scale cell subtypes in the tumor microenvironment

**Figure 3:**
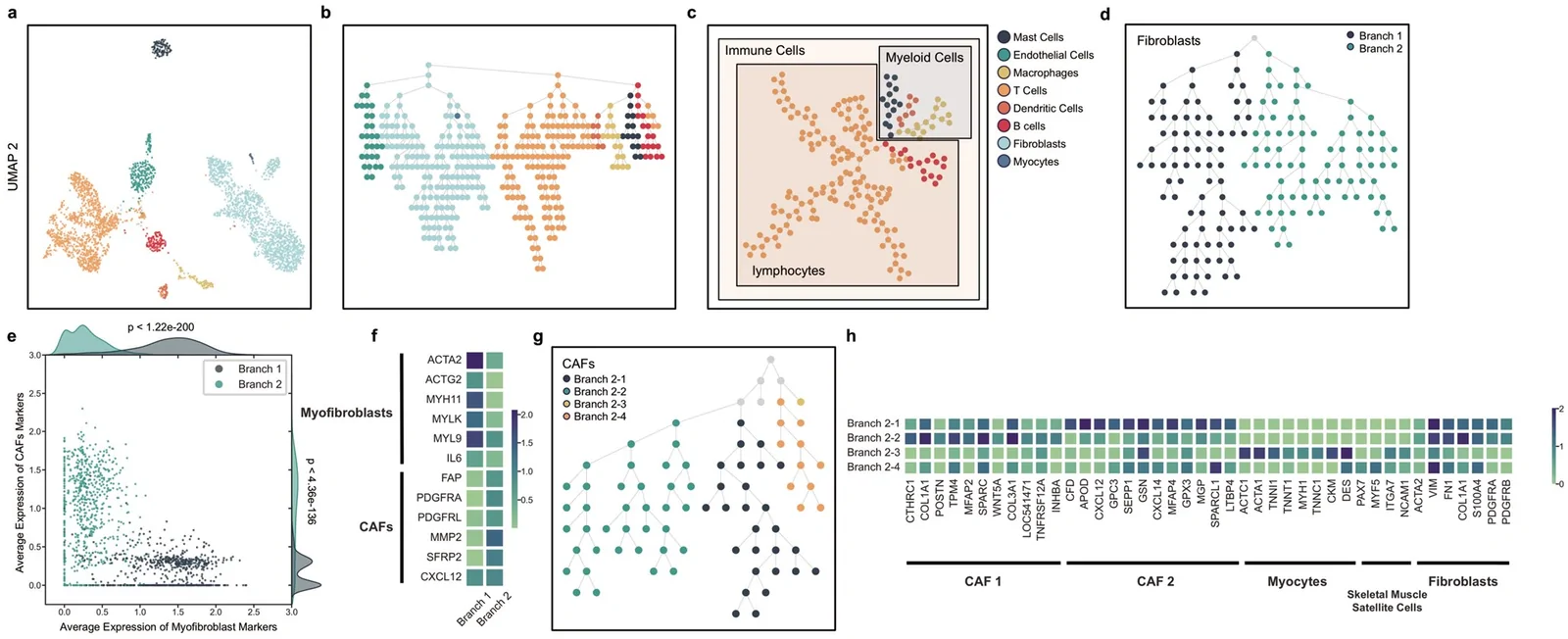
Multi-scale hierarchical analysis of tumor microenvironment (TME) cellular functional diversification using scMustree in the Puram dataset. (a) UMAP projection of single-cell transcriptomes color-coded by annotated cell types. (b) The complete multi-scale hierarchy constructed by scMustree, with node colors denoting dominant cell type annotations. (c) Flattened representation of the immune-cell-dominated sub-hierarchy, with node colors distinguishing dominant immune cell types. (d) Fibroblast-dominated sub-hierarchy with dual-color highlighting of its two principal branches. Branch 1 (marker genes: ACTA2, MYH11, MYL9) and Branch 2 (marker genes: FAP, PDGFRA, MMP2). (e) Joint plot showing branch-specific expression patterns of myofibroblast and CAF markers across single cells (central scatter plot) with marginal density distributions. Statistical significance is assessed using a two-sided Wilcoxon rank sum test, with *P*-value adjustment via Bonferroni correction based on the total number of genes. (f) Comparative visualization of mean marker expression levels between branches for myofibroblasts and CAFs. (g) CAF-dominated sub-hierarchy with quad-color differentiation of its four major branches. Branch 2-1 (marker genes: APOD, CXCL12), Branch 2-2 (marker genes: COL1A1, COL3A1), Branch 2-3 (marker genes: DES, ACTA1), and Branch 2-4 (marker genes: S100A4, VIM, PAX7). (h) Comparative visualization of mean marker expression levels between branches for five cell types.

Transcriptomic profiling has revolutionized our understanding of regulatory programs and disease subtypes across human malignancies (Chen *et al.* 2020). Emerging evidence highlights that functional diversification of non-malignant cells within the tumor microenvironment (TME) critically influences tumor progression and therapeutic responses (Kürten *et al.* 2021). To investigate this cellular complexity, we analyze the Puram dataset comprising 3363 non-malignant cells from 18 head and neck squamous cell carcinoma (HNSCC) patients (Puram *et al.* 2017). Initial clustering categorizes these cells into 8 major types: T cells, B/plasma cells, macrophages, dendritic cells, mast cells, endothelial cells, fibroblasts, and myocytes. The spatial relationships of these types are visualized through UMAP dimensionality reduction (Fig. 3a).

Applying scMustree to this dataset reconstructs a multi-scale hierarchical architecture that comprehensively maps functional relationships between cell types across multiple scales (Fig. 3b). This architecture aligns with established cell type annotations, as evidenced by the distinct expression patterns of markers in branch-specific nodes (Fig. S4, available as supplementary data at *Bioinformatics* online; marker lists in Table S4, available as supplementary data at *Bioinformatics* online). Beyond validating established classifications, the hierarchical topology reveals functional relationships and intra-population heterogeneity. For instance, macrophages and dendritic cells occupy neighboring branches due to shared myeloid lineage characteristics, and the fibroblast-dominated sub-hierarchy exhibits spatial segregation corresponding to molecularly distinct subtypes. The differential expression levels of fibroblast markers within its branches point to the existence of functionally distinct subtypes.

To further validate the topology of this hierarchy, we first examine the immune-cell-dominated sub-hierarchy in its flattened representation (Fig. 3c). The structure separates lymphoid lineages (T cells, B/plasma cells) from myeloid lineages (macrophages, dendritic cells, mast cells), consistent with established immunological hierarchies. Given the emerging clinical significance of tumor-associated fibroblasts, subsequent analysis focuses on the fibroblast-dominated sub-hierarchy (Fig. 3d). Through differential expression analysis, scMustree identifies two major fibroblast subtypes originally described in the dataset: myofibroblasts (Branch 1) characterized by elevated *ACTA2*, *MYH11*, and *MYL9* expression, and cancer-associated fibroblasts (CAFs, Branch 2) marked by *FAP*, *PDGFRA*, and *MMP2* expression. The detailed analytical pipeline and statistical thresholds for the identification of differential genes are provided in Supplementary Note 3, available as supplementary data at *Bioinformatics* online. A scatter plot with marginal distributions visualizes the average expression levels of these subtype-specific markers across individual cells, color-coded by branch (Fig. 3e), with comparative average expression levels of each marker between branches in Fig. 3f.

To delineate finer functional subdivisions within CAFs, we perform progressive branching analysis along the CAF sub-hierarchy, which reveals four distinct sub-branches (Branches 2–1 to 2–4, Fig. 3g). The average expression levels of each marker in these branches are shown in Fig. 3h. Differential expression analysis identifies Branches 2–1 and 2–2 as corresponding to previously reported CAF1/CAF2 subtypes. Cells in Branch 2–3 exhibit myocyte-like features (*DES*${\;}^{+}$, *ACTA1*${\;}^{+}$) which are previously annotated as myocytes. Notably, after merging with Branch 2–4, this composite branch localizes adjacent to the fibroblast-dominated branches (Branches 2–1 and 2–2). Further characterization reveals that Branch 2–4 cells co-express fibroblast markers (*S100A4*${\;}^{+}$, *VIM*${\;}^{+}$) and muscle satellite cell markers (*PAX7*${\;}^{+}$), suggesting they may represent activated muscle satellite cells with increased gene expression related to extracellular matrix remodeling and fibrosis processes, representing a previously unrecognized cell population in the original study. Building on this multi-scale hierarchy, scMustree similarly enables multi-scale analysis of T cell heterogeneity. Through differential expression analysis and the analysis of the average expression of markers, we confirm that its four main branches match four reported subtypes: regulatory T cells, conventional *CD4*${\;}^{+}$ T helpers, and two cytotoxic *CD8*${\;}^{+}$ T cell states (activated and exhausted) (Fig. S5, available as supplementary data at *Bioinformatics* online).

In summary, the multi-scale hierarchy constructed by scMustree not only captures the multi-scale type relationships obtained through multiple rounds of analysis in the original study but also uncovers novel TME-associated cell types. This demonstrates its utility for decoding functional diversity in complex environments.

### 3.3 scMustree identifies Alzheimer’s-related microglial subtypes in the human hippocampus

**Figure 4:**
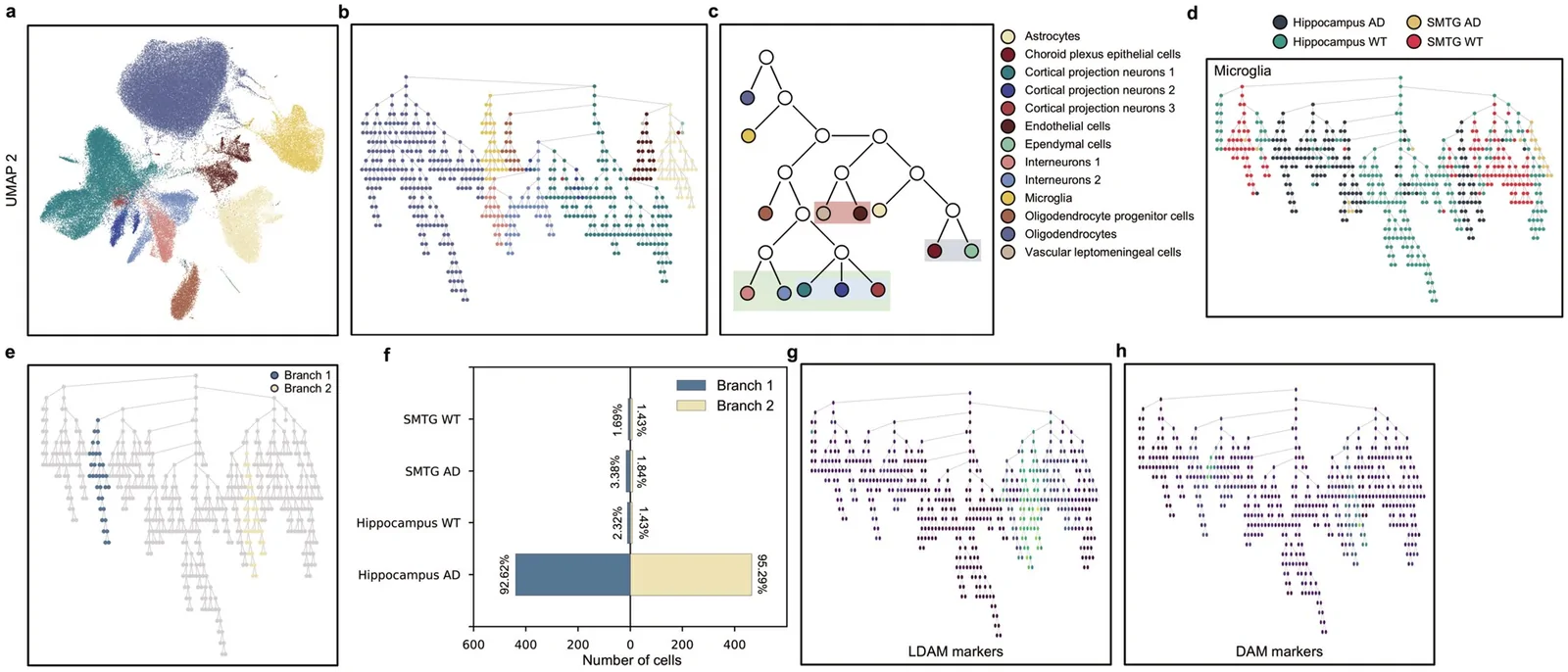
Analysis of cell type relationships and functional diversification in the Alzheimer dataset using scMustree. (a) UMAP projection of single-cell transcriptomes, color-coded by annotated cell types. (b) The complete multi-scale hierarchy is constructed by scMustree, with node colors denoting dominant cell type annotations. (c) Key branching patterns in the refined topology of scMustree’s hierarchy. (d) Microglia-specific hierarchy structure constructed by scMustree with cell sources (AD/WT in hippocampus/SMTG). (e) Microglia sub-hierarchy highlighting two AD hippocampal-enriched branches. (f) Comparative cell source composition in microglial sub-branches from AD hippocampus. (g, h) Expression levels of specific markers for LDAM (g) and DAM (h) in the Alzheimer dataset across different hierarchical nodes. The color intensity of each node reflects the relative expression level of the marker.

The diversity in the functions of brain cell types is of great significance for gaining a deeper understanding of neurodegenerative diseases such as Alzheimer’s disease (AD). Leveraging an Alzheimer dataset comprising 1 18 240 cells across 13 cell types from human brain samples (hippocampus and superior/middle temporal gyrus (SMTG)) of six AD patients and six matched controls, we apply scMustree to investigate cellular dynamics (Sziraki *et al.* 2023). The spatial relationships among cell types are visualized through UMAP dimensionality reduction (Fig. 4a).

scMustree constructs a multi-scale hierarchical architecture that captures cell type relationships (Fig. 4b), with refined topological analysis showcasing its capacity to identify adjacent branches of cell types that may reflect functional modules potentially linked to AD pathogenesis (Fig. 4c). Specifically, three cortical projection neuron subsets (1, 2, and 3) and two interneuron subsets (1 and 2) are adjacent after each being merged into their respective main branches. Cortical projection neurons and interneurons form adjacent branches, reflecting their complementary roles in neural circuit balance. Vascular leptomeningeal cells and endothelial cells are grouped together, consistent with their shared involvement in blood-brain barrier function—a key pathological axis in AD (Gold *et al.* 2007, Van De Haar *et al.* 2016). Similarly, choroid plexus epithelial cells and ependymal cells are adjacent, both participating in cerebrospinal fluid circulation and waste clearance, processes implicated in Aβ accumulation (Erickson *et al.* 2012).

Given the complex and multifaceted role of microglia in Alzheimer’s disease (AD), we used scMustree to reconstruct their developmental hierarchy. We then examine the distribution of cells from Alzheimer’s patients (AD) and wild-type (WT) controls across this microglia-specific hierarchical architecture (Fig. 4d). Combining the distribution of cells, we find two main branches of microglia composed of cells in the hippocampus of AD patients, Branch 1 and Branch 2, accounting for 92.62% and 95.29% respectively (Figs 4e and f). To explore their potential relevance to AD, we conduct differential gene expression analysis and GO enrichment analysis to analyze their functions which show distinct functional profiles, highlighting their potential roles in the disease. The detailed procedures for the Gene Ontology functional enrichment analysis are presented in Supplementary Note 4, available as supplementary data at *Bioinformatics* online. Branch 1 is mainly characterized by the high expression of heat shock protein family genes (such as *HSP90AA1*, *HSPH1*, *HSPA1A*) and the lysosomal sorting receptor *SORL1*, which is significantly enriched in the negative regulation pathways of the unfolded protein response (UPR) and inclusion body assembly. As for Branch 2, it shows characteristics closely related to metabolism and inflammation. Its highly expressed genes, such as *ACSL1*, *HIF1A*, and *SPP1*, drive hypoxia-induced glycolytic reprogramming and the activation of pro-inflammatory signals. This metabolic reprogramming may lead to the accumulation of lipid droplets, which is closely related to intracellular lipid metabolic disorders. Therefore, the microglia of Branch 2 may promote the accumulation of lipid droplets through these metabolic changes, thereby affecting cell function and the pathological process of AD. Interestingly, we find that this subtype is very similar to the lipid-associated *ACSL1*${\;}^{+}$ microglia subtype reported by Haney *et al.* (2024) They report LD-accumulating microglia (LDAM), a state of disease-associated microglia (DAM) microglia, which co-expresses more metabolic state regulators such as *NAMPT* and *DPYD*. We visualize the expression distribution of LDAM and DAM-related markers in the hierarchical architecture, further confirming their identities (Fig. 4g and h).

In conclusion, scMustree can not only comprehensively reflect the relationships among cell types in disease states but also display disease-related cell type subtypes, which play different functions in the disease, thus providing further insights for disease analysis.

## 4 Discussion

In this study, we present scMustree, a computational framework that overcomes the limitations of conventional clustering by inferring multi-scale hierarchies that capture both global and local heterogeneity. scMustree advances single-cell analysis by treating clustering as a dynamic hierarchical discovery process rather than static partitioning. Through iterative cluster decomposition and biologically informed merging strategies, scMustree generates high-purity cell clusters (Supplementary Note 5, available as supplementary data at *Bioinformatics* online) and establishes functional relationships between cell types. By identifying differentially expressed genes (DEGs) for each cluster and constructing ensemble decision trees based on their expression patterns, scMustree defines cluster-specific functional rankings (CFR). These CFR guide hierarchical merging and offer insights into the expression dynamics of cluster-specific genes.

To evaluate the contribution of individual components, we perform an ablation study comparing the full scMustree model with three variants that removed iterative decomposition, replaced the CFR-based RBO similarity with Euclidean distance, or substituted RBO with Kendall’s tau (Supplementary Note 6; Tables S5 and S6, available as supplementary data at *Bioinformatics* online). The full model consistently outperforms all variants. The CFR-based RBO similarity contributes the largest performance gain, substantially surpassing conventional pseudobulk distance measures. The CFR representation itself also provides meaningful information beyond simple expression proximity, as the Kendall’s tau variant—which retains CFR but replaces RBO—outperforms the Euclidean-based variant. Furthermore, while a single clustering round suffices for relatively simple datasets, the iterative decomposition strategy proves particularly beneficial for resolving complex and deeply structured cellular hierarchies. Collectively, these results confirm that the performance advantages of scMustree arise from the synergistic combination of its core components, with the functional ranking-based similarity metric serving as the primary driver.

The framework’s branch-level type discrimination and accurate representation of biological relationships further validate its utility for constructing multi-scale cellular hierarchies. Beyond the applications in the tumor microenvironment and Alzheimer’s disease discussed above, scMustree has been further validated across diverse biological scenarios, including evolutionarily conserved cell-type relationships in glaucoma, patient-specific pathological subtypes in Alzheimer’s disease, novel cellular subtypes in dendritic cells and retinal bipolar cells, and spatial organization principles during mouse embryonic development. Detailed results are provided in Supplementary Notes 7–10, available as supplementary data at *Bioinformatics* online.

The successful application of scMustree to various diseases highlights its potential as a valuable tool for biomedical research. By enabling deeper understanding of cellular heterogeneity and functional diversity, scMustree facilitates the identification of disease-specific cell states and their associated molecular mechanisms, potentially guiding targeted therapies and improved diagnostic strategies. The framework’s integration of multi-scale and multi-resolution analyses makes it applicable to a wide range of biological systems, from developmental biology to immunology and neuroscience. Scalability analysis on a PBMC dataset of up to 68 579 cells further confirms that scMustree’s runtime scales with the number of terminal populations rather than total cell count (Supplementary Note 11, available as supplementary data at *Bioinformatics* online), supporting its application to datasets exceeding one hundred thousand cells.

In conclusion, scMustree provides a robust and comprehensive framework for decoding functional diversity within complex tissues, enabling researchers to explore intricate cellular landscapes underlying biological processes and diseases.

## Supplementary Material

btag672_Supplementary_Data

## Data Availability

The details of the datasets used in this study are reported in Table S1, available as supplementary data at *Bioinformatics* online. All described datasets are obtained from various public databases and can be accessed using the accession numbers provided in Table S1, available as supplementary data at *Bioinformatics* online. These databases include the NCBI Gene Expression Omnibus (GEO) https://www.ncbi.nlm.nih.gov/geo/ and the Single Cell Portal https://singlecell.zendesk.com/hc/en-us. The Dendritic cell dataset from the 68k PBMC dataset is obtained from the website of 10X genomics (https://www.10xgenomics.com/datasets/fresh-68-k-pbm-cs-donor-a-1-standard-1-1-0). The Bipolar cell dataset from the mouse retina dataset is available on GitHub https://github.com/OSU-BMBL/marsgt/tree/main/Data. Source data are provided in this paper.
