# Supplementary material for: scMustree: a multi-scale functional hierarchy for single-cell transcriptomic analysis
Source: Bioinformatics. 2026 Sep 10;42(9):btag672. doi: 10.1093/bioinformatics/btag672 (PMC13614420; doi:10.1093/bioinformatics/btag672)
Supplement: Supplementary file 1 [file btag672_supplementary_data.pdf]

Supporting Information

scMustree: a multi-scale functional hierarchy for single-cell transcriptomic analysis

Yunpei Xu, Shaokai Wang, Liqing Ding, Hongdong Li, and Jianxin Wang**

Supplementary Figures

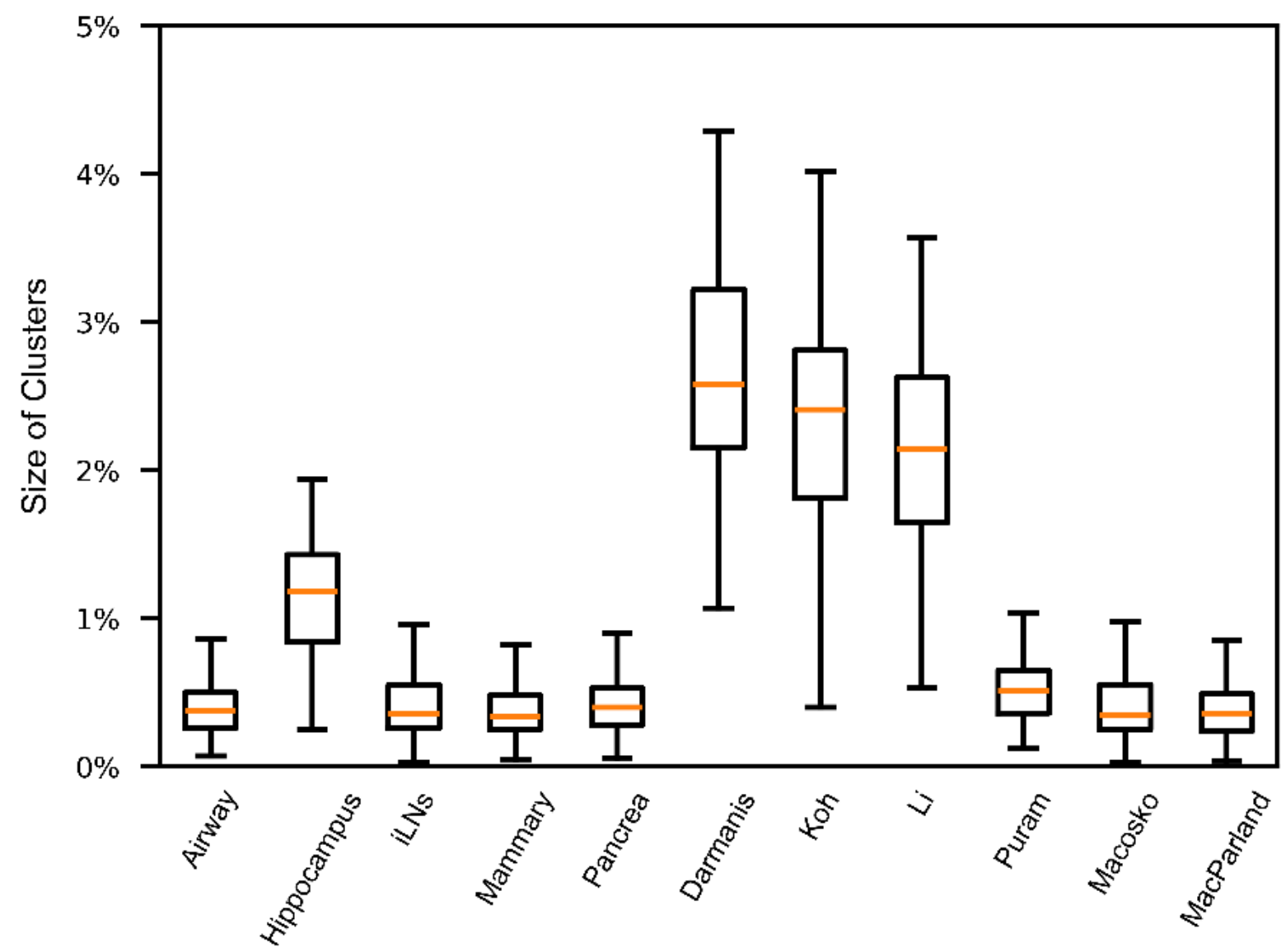

Figure S1. The size distribution of the terminal populations (decomposed clusters) within the hierarchies constructed by scMustree across the benchmarking datasets. Boxes extend from the first to the third quartile (Q1 to Q3) with a line in the middle that represents the median. Lines extending from both ends of the box indicate variability outside Q1 and Q3. The minimum/maximum whisker values are calculated as Q1/Q3 $-/+ \mathbf{1.5} \times$ IQR.

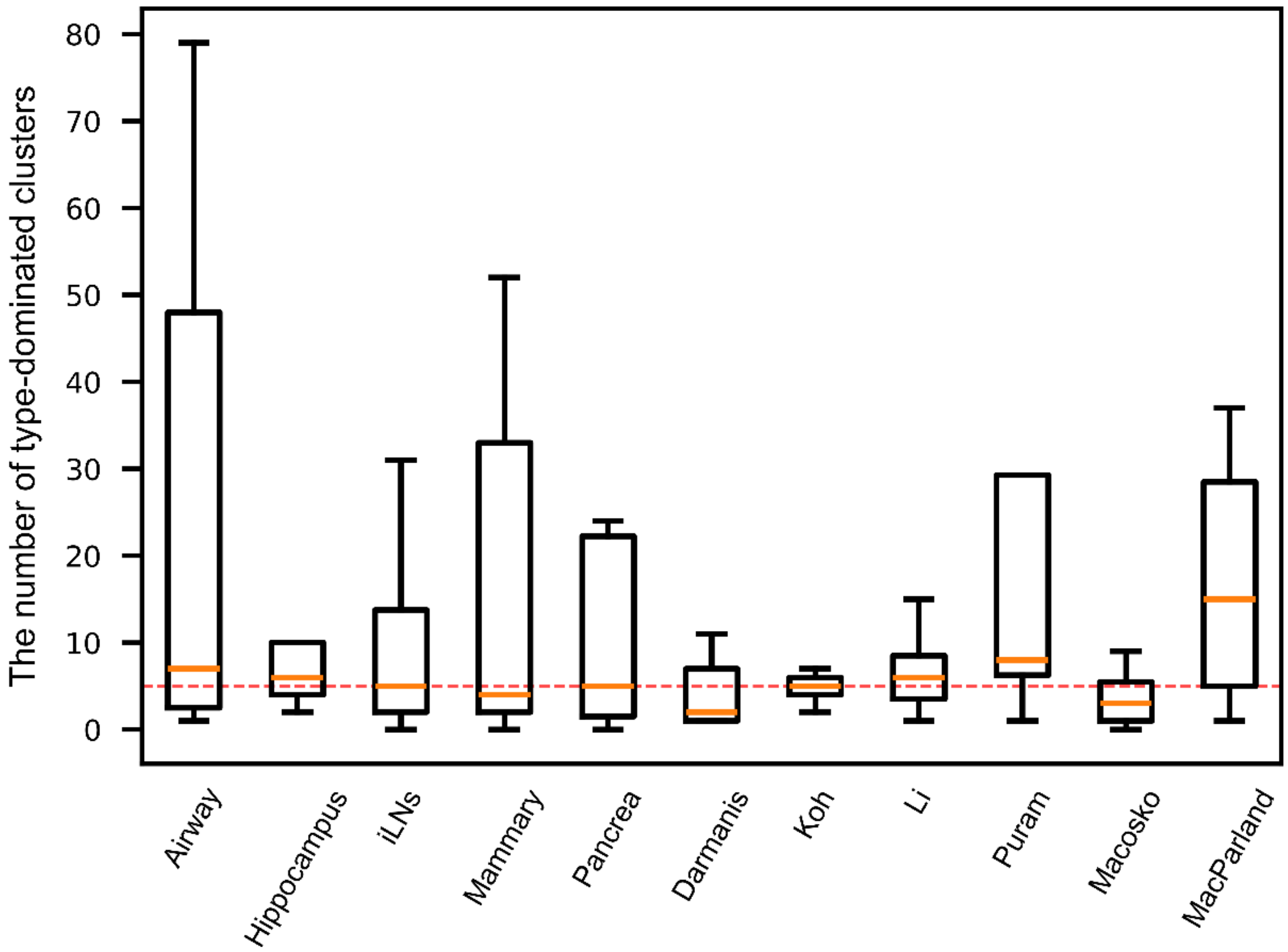

Figure S2. The distribution of the number of terminal populations (decomposed clusters) within the hierarchies constructed by scMustree dominated by different types across the benchmarking datasets. The red dashed line represents that the number of clusters is 5. Boxes extend from the first to the third quartile (Q1 to Q3) with a line in the middle that represents the median. Lines extending from both ends of the box indicate variability outside Q1 and Q3. The minimum/maximum whisker values are calculated as Q1/Q3 $-/+ \mathbf{1.5} \times$ IQR.

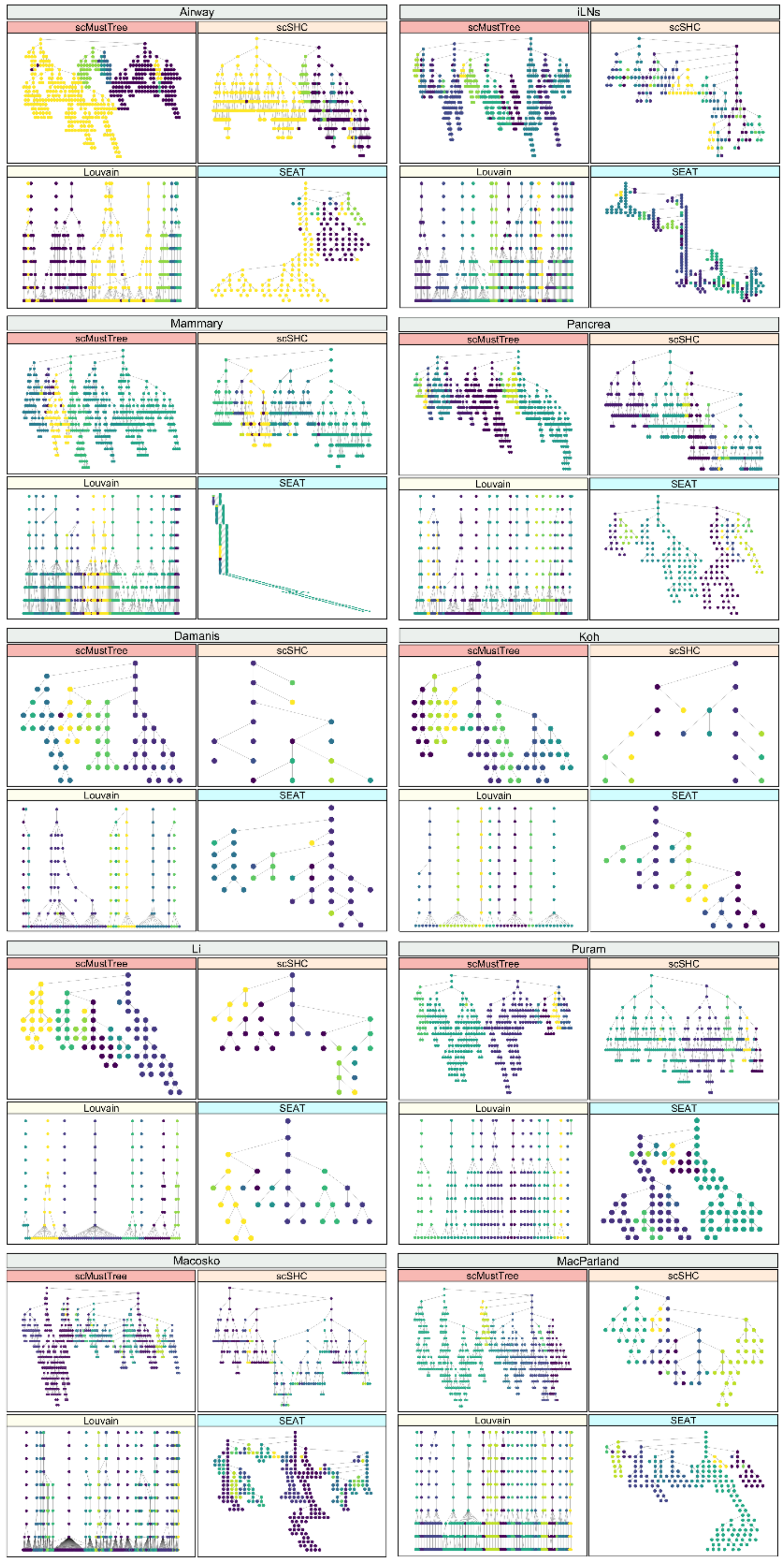

Figure S3. Cell type-resolved hierarchies generated by four hierarchical methods in 10 single cell RNA seq datasets. Each node's color corresponds to the dominant cell type within it, providing a clear visual representation of the cell type distribution in the constructed hierarchies.

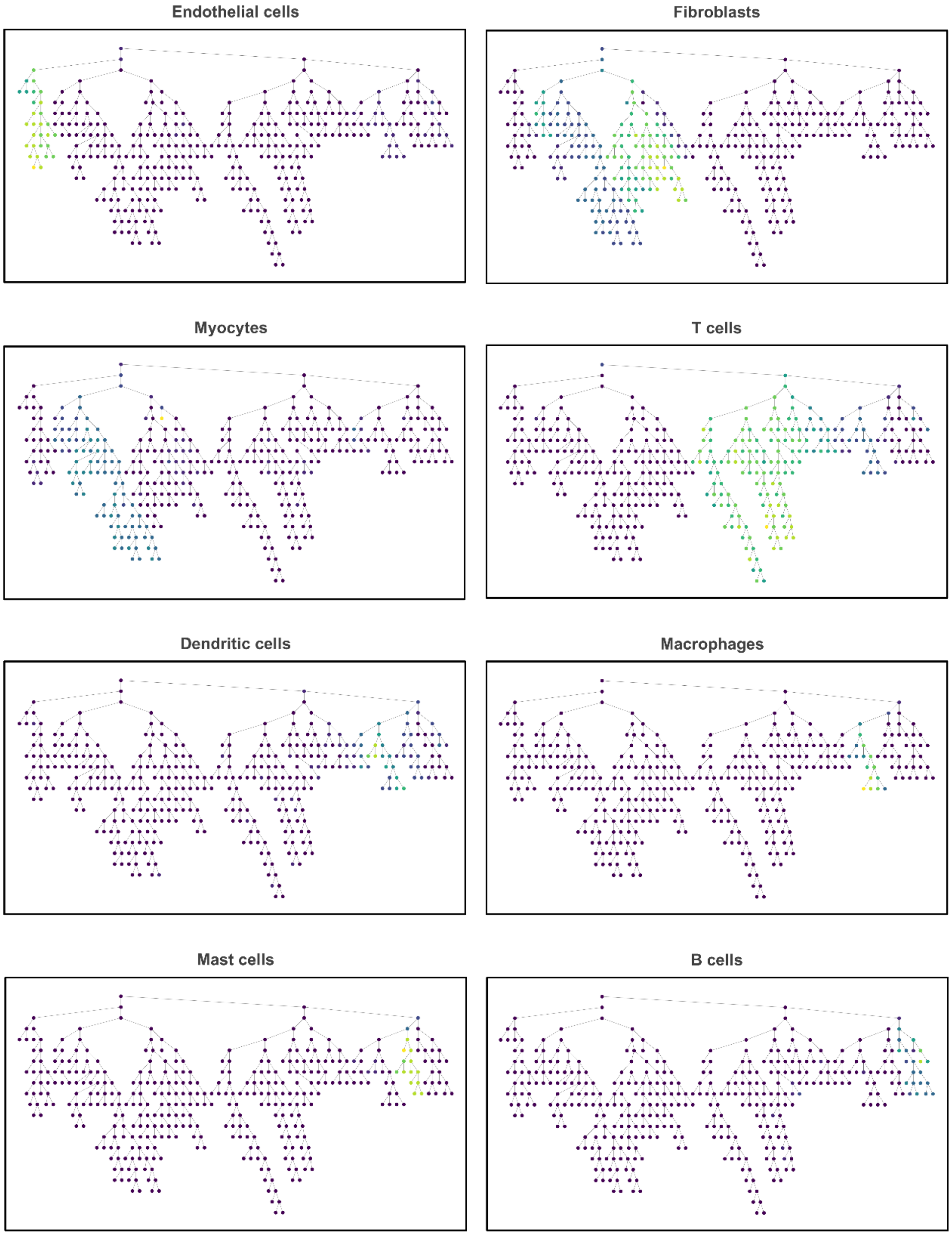

Figure S4. Expression levels of specific markers for 8 major cell types in the Puram dataset across different hierarchical nodes. The color intensity of each node reflects the relative expression level of the marker.

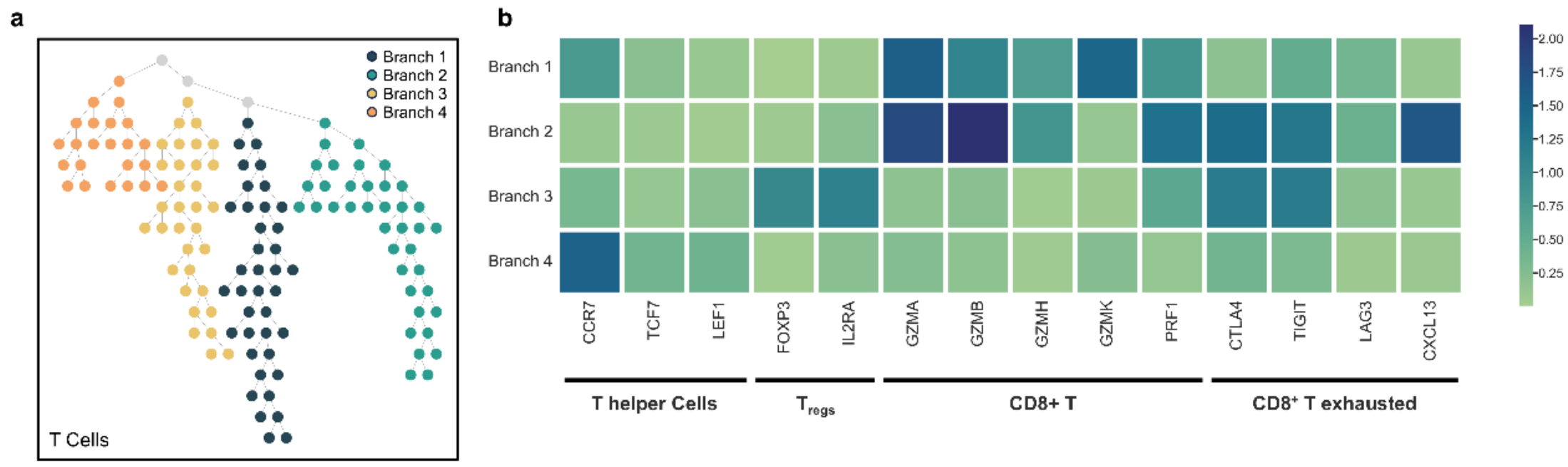

Figure S5. Progressive branching analysis along the T-cell sub-hierarchy through scMustree-derived hierarchical architecture to delineate finer functional subdivisions. (a) T-cell-dominated sub-hierarchy with quad-color differentiation of its four major branches. (b) Comparative visualization of mean marker expression levels between branches for four T-cell subtypes.

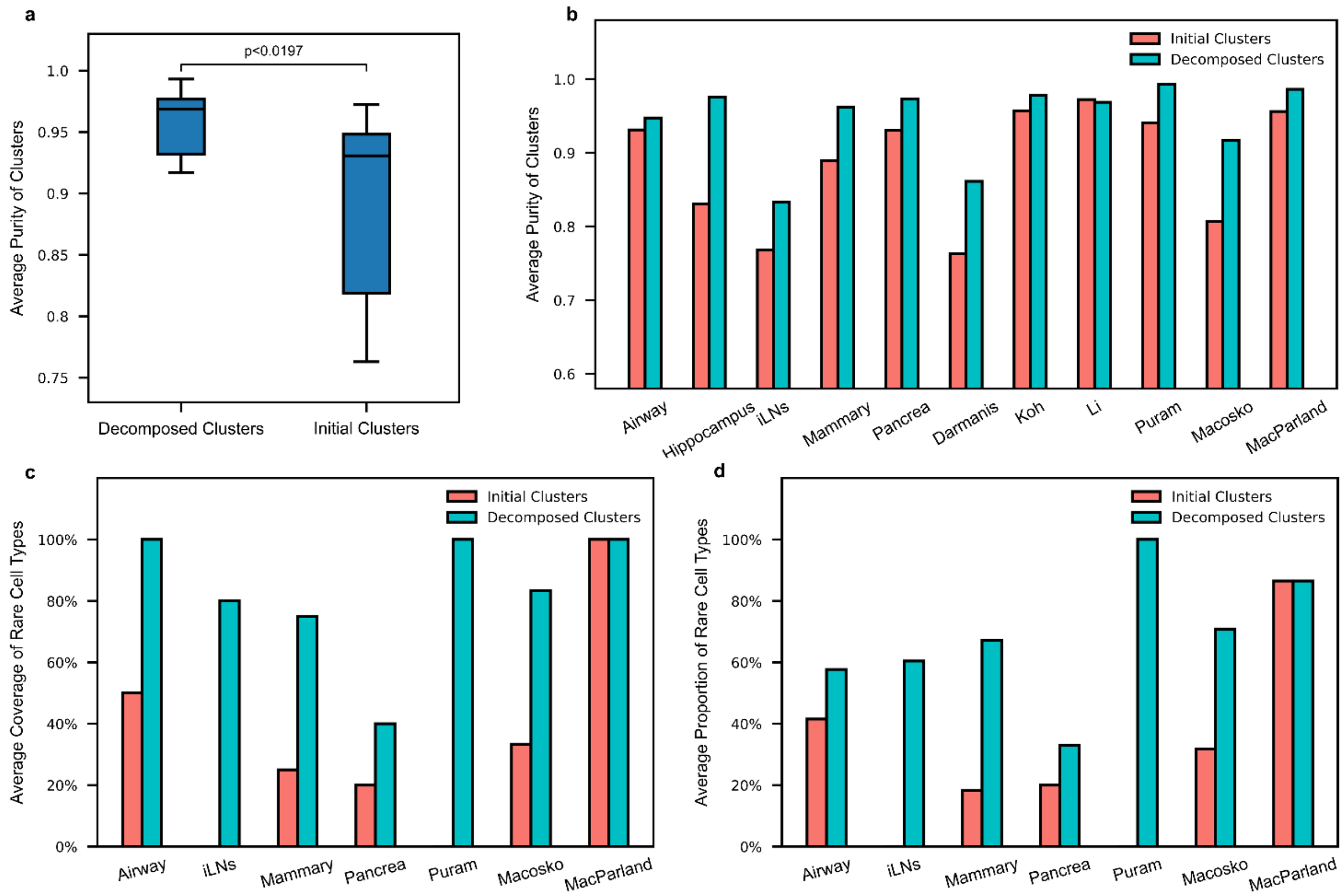

Figure S6. Benchmarking scMustree's performance in terminal population determination. (a) Distribution of average cluster purity before and after decomposition. (b) Average cluster purity for each dataset before and after decomposition. (c) Average coverage of rare cell types before and after decomposition. (d) The average proportion of cells involved in rare cell types before and after decomposition.

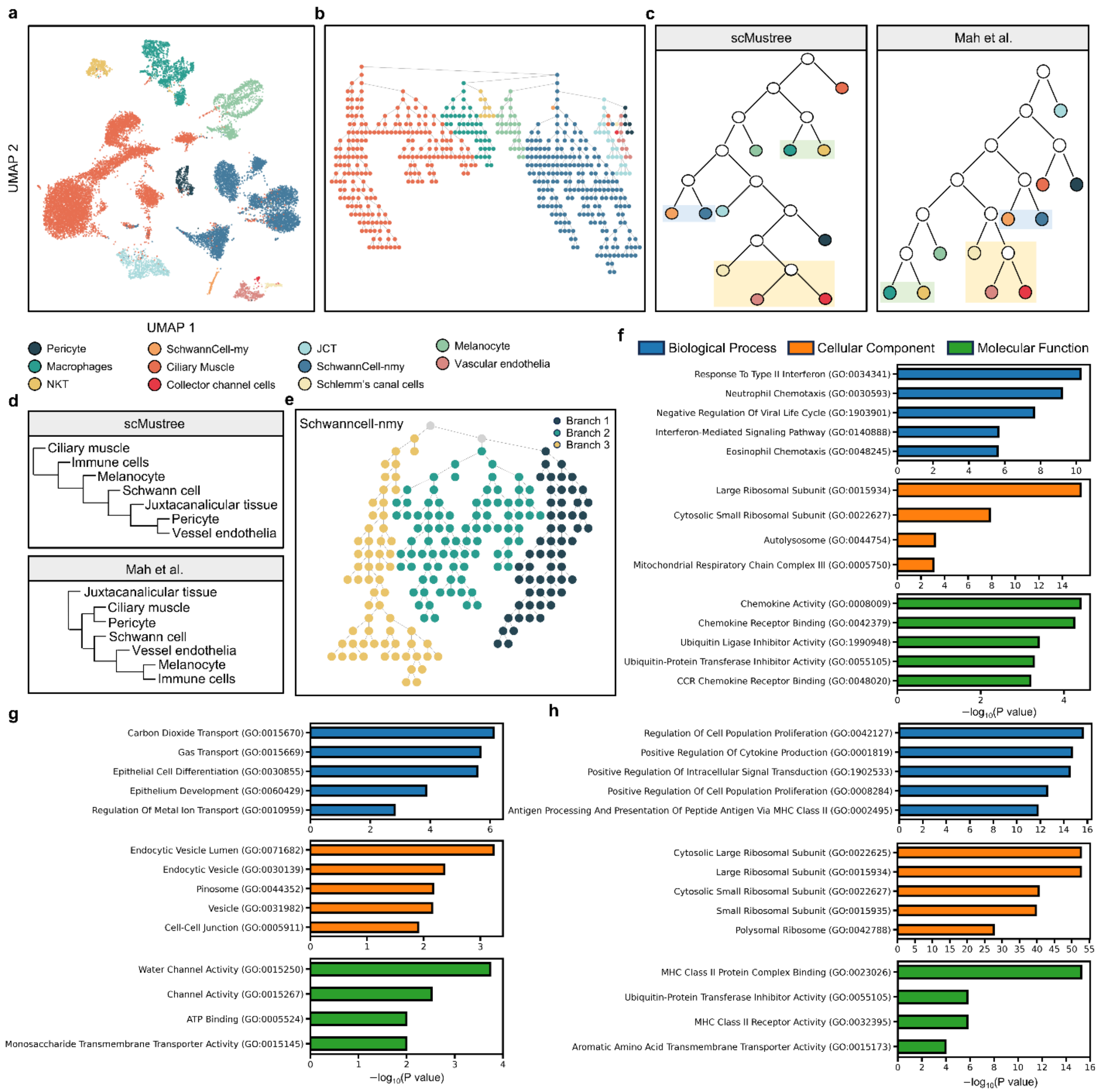

Figure S7. Analysis of cell type relationships and functional diversification in the human glaucoma dataset using scMustree. **(a)** UMAP projection of single-cell transcriptomes, color-coded by annotated cell types. **(b)** The complete hierarchical architecture constructed by scMustree, with node colors denoting dominant cell type annotations. **(c)** Comparison of key branching patterns between scMustree and Mah et al.'s cross-species cell phylogeny in the refined topology. **(d)** Topological distinctions in inter-type relationships after integration of functionally related cell types, comparing scMustree with Mah et al.'s architecture. **(e)** Non-myelinating Schwann cell-dominated sub-hierarchy, with three colors highlighting its three principal branches. **(f~h)** GO enrichment analysis results correspond to Branch 1 **(f)**, Branch 2 **(g)**, and Branch 3 **(h)**. NKT, Natural Killer T cells; my, myelinating; nmy, non-myelinating; JCT, juxtacanalicular canal tissue.

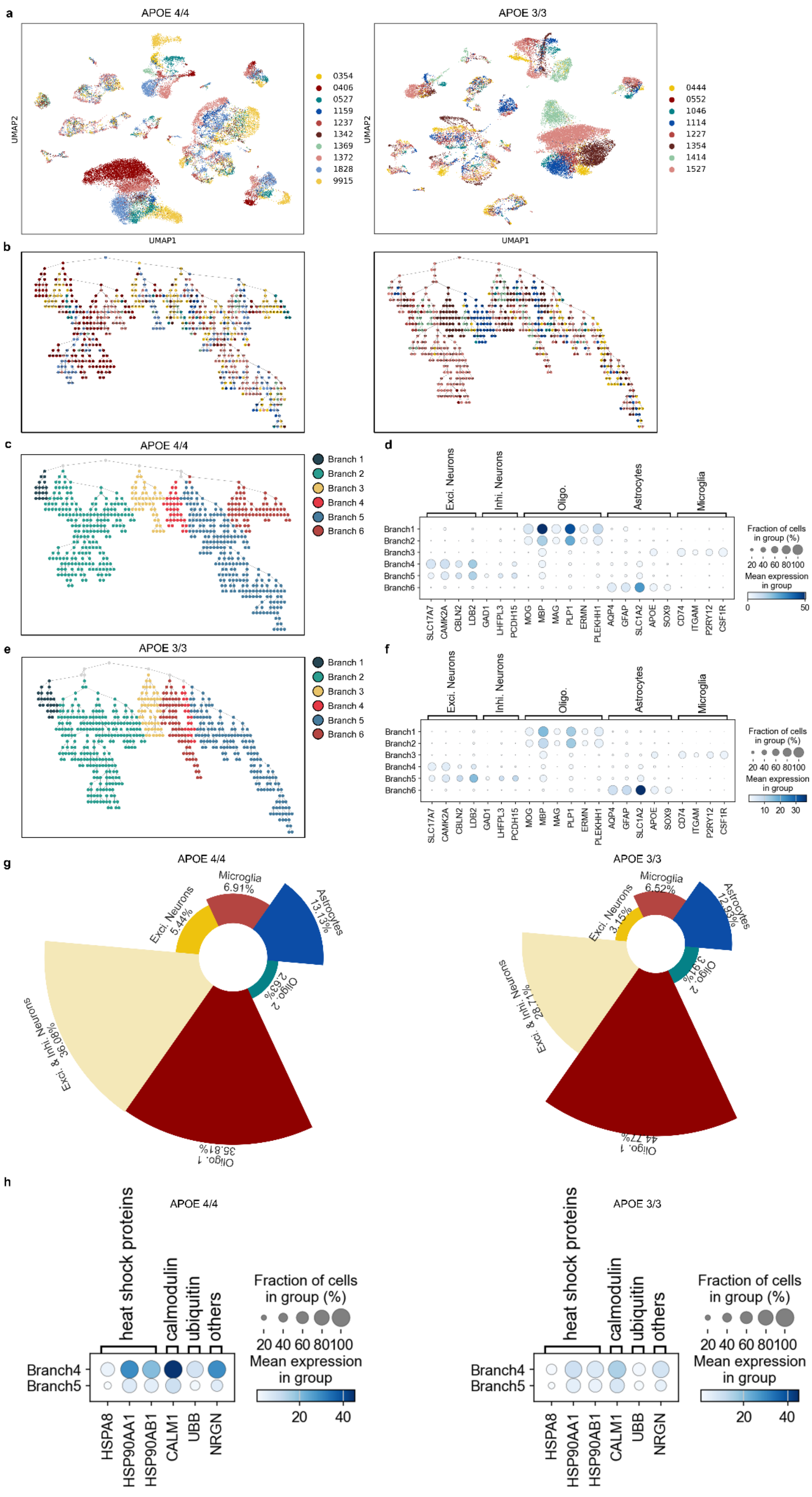
a
APOE 4/4
APOE 3/3
UMAP1
UMAP2
0354
0406
0527
1159
1237
1342
1369
1372
1828
9915
0444
0552
1046
1114
1227
1354
1414
1527
b
c
APOE 4/4
Branch 1
Branch 2
Branch 3
Branch 4
Branch 5
Branch 6
d
Exci. Neurons
Inhi. Neurons
Oligo.
Astrocytes
Microglia
Branch1
Branch2
Branch3
Branch4
Branch5
Branch6
SLC17A7
CAMK2A
CBLN2
LDB2
GAD1
LHFPL3
PCDH15
MOG
MBP
MAG
PLP1
ERMN
PLEKHH1
AQP4
GFAP
SLC1A2
APOE
SOX9
CD74
ITGAM
P2RY12
CSF1R
Fraction of cells in group (%)
20 40 60 80100
Mean expression in group
0
50
e
APOE 3/3
f
10 20 30
g
APOE 4/4
Microglia 6.91%
Astrocytes 13.13%
Exci. Neurons 5.44%
Oligo. 2 2.63%
Exci. & Inhi. Neurons 36.08%
Oligo. 1 35.81%
APOE 3/3
Microglia 6.52%
Astrocytes 12.93%
Exci. Neurons 3.15%
Oligo. 2 3.91%
Exci. & Inhi. Neurons 28.71%
Oligo. 1 44.77%
h
APOE 4/4
APOE 3/3
heat shock proteins
calmodulin
ubiquitin
others
Branch4
Branch5
HSPA8
HSP90AA1
HSP90AB1
CALM1
UBB
NRGN
Fraction of cells in group (%)
20 40 60 80100
Mean expression in group
20
40

Figure S8. Independent analysis of two APOE genotypes in AD patients. **(a)** UMAP projections of single-cell transcriptomes for each genotype, color-coded by sample ID. **(b)** Complete hierarchical architecture constructed by scMustree with node colors indicating cell sample origins. **(c)** scMustree-generated hierarchy for APOE 4/4 genotype patients. Major branches are distinguished using distinct colors. **(d)** Expression patterns of cell type-specific markers across major branches. **(e)** scMustree-generated hierarchy for APOE 3/3 genotype patients. Major branches are distinguished using distinct colors. **(f)** Expression patterns of cell type-specific markers across major branches. **(g)** Comparison of cell type composition proportions across branches between both hierarchies. **(h)** Gene expression patterns (major heat shock proteins, calmodulin, ubiquitin B) in neuron-dominant branches.

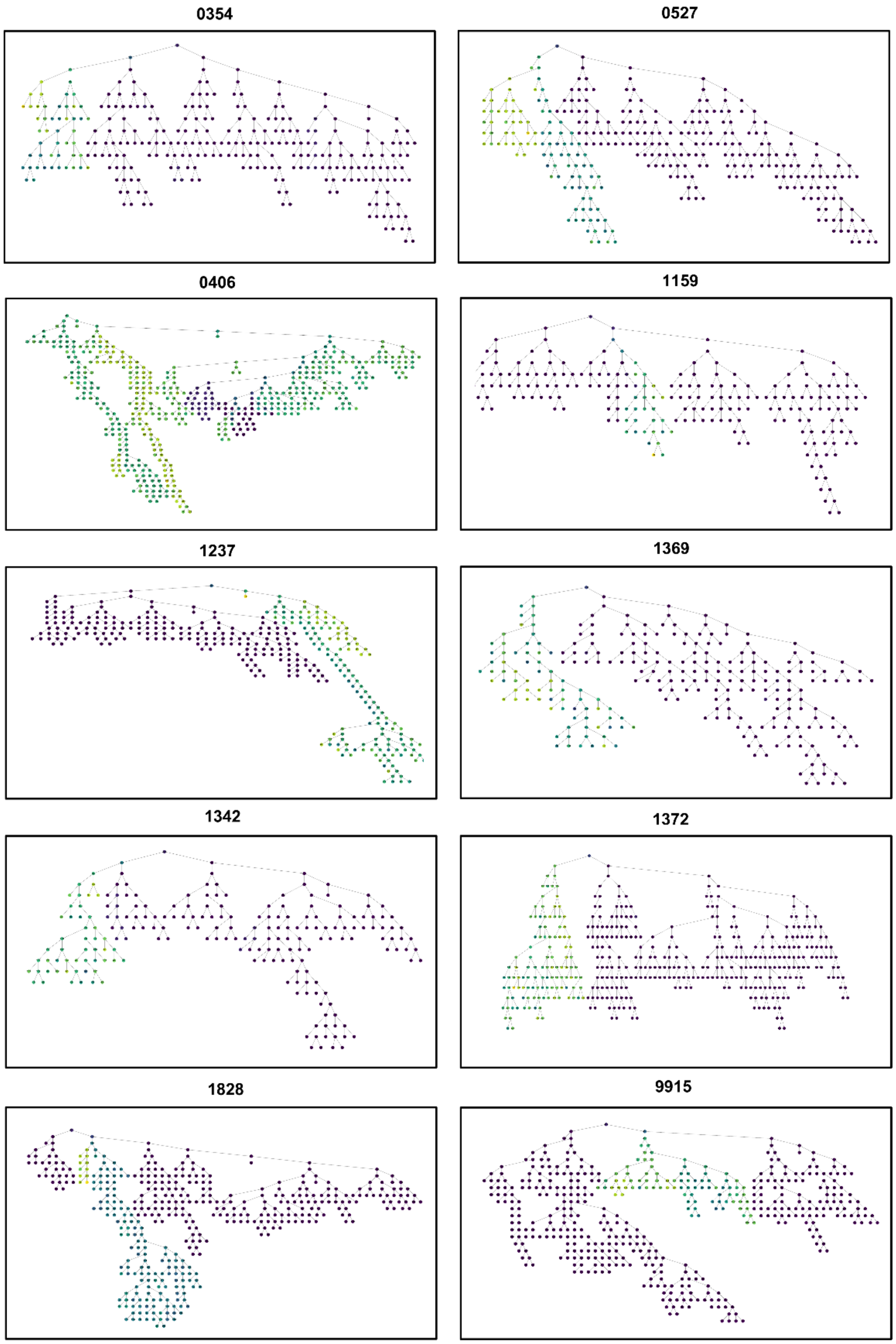

Figure S9. Expression levels of oligodendrocyte-specific markers across distinct hierarchical nodes within the dendrograms derived from different Alzheimer's disease patients. The color intensity of each node reflects the relative expression level of the marker.

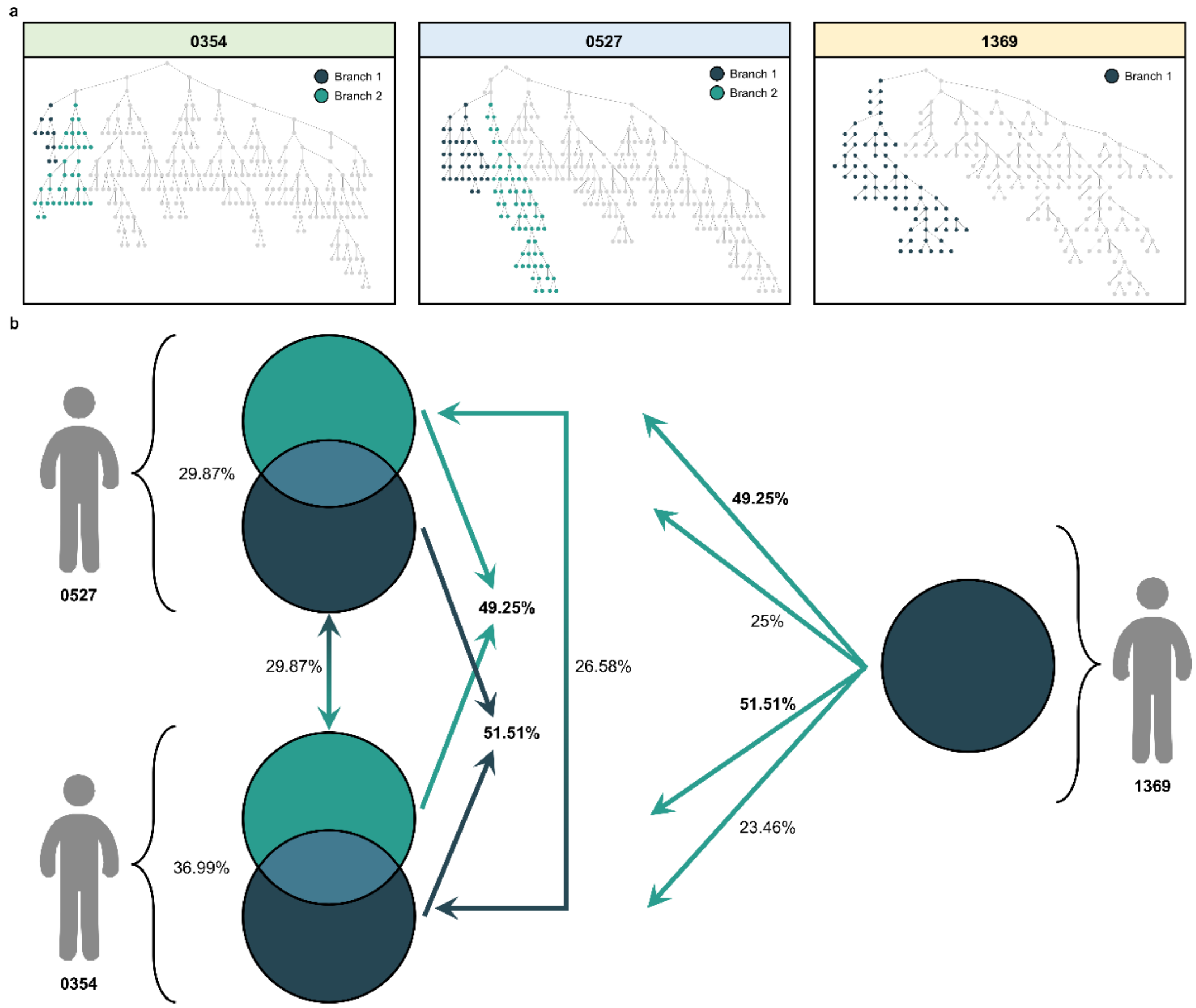

Figure S10. Individual analysis of three APOE 4/4 AD patients. **(a)** Complete hierarchical architectures constructed by scMustree for each patient. Oligodendrocyte branches are color-coded, revealing distinct branching patterns. **(b)** Jaccard similarity comparisons of the top 50 differentially expressed genes (DEGs) between branches.

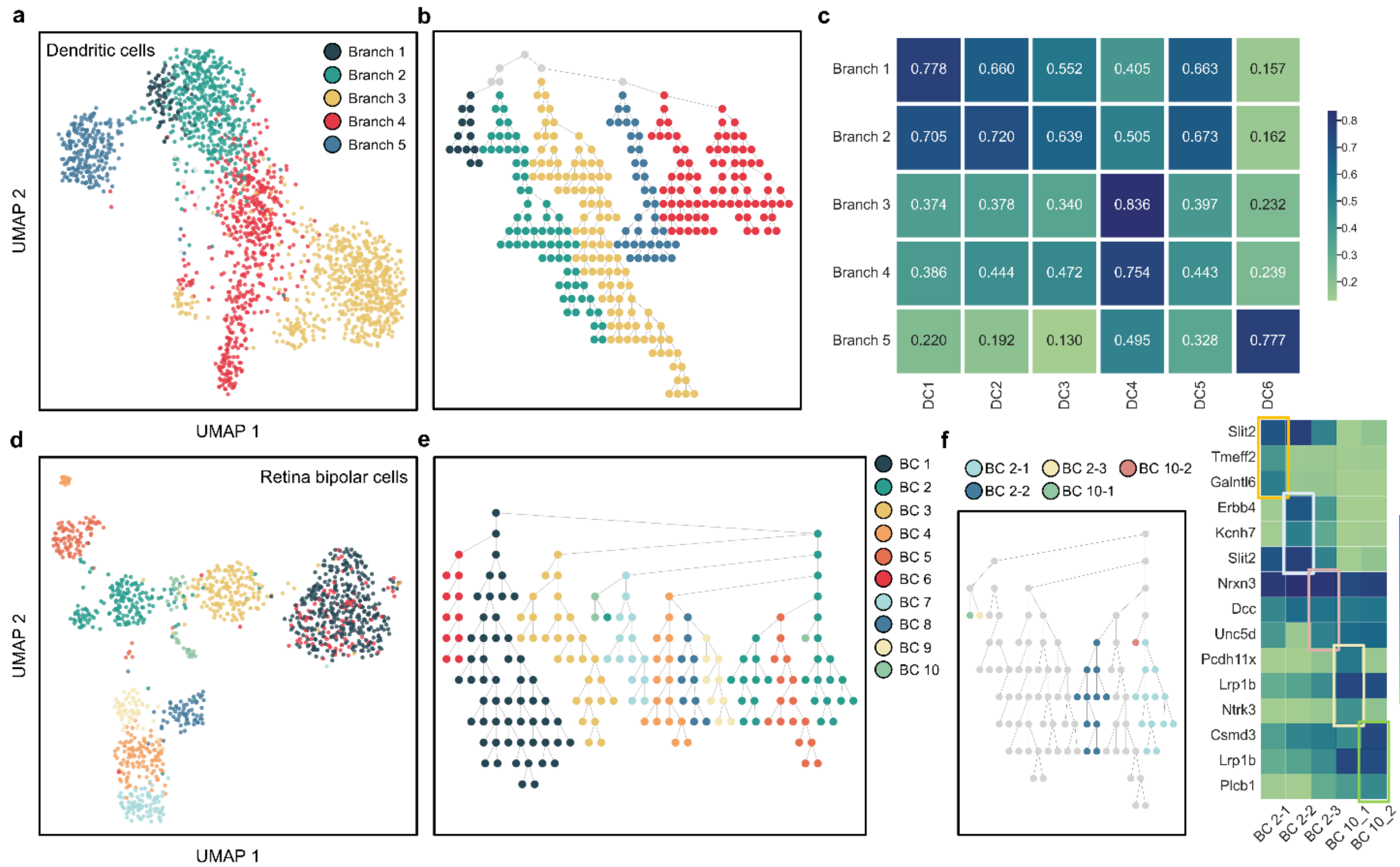

Figure S11. Analysis of dendritic cells (DCs) in human PBMCs and bipolar cells (BCs) in mouse retinal datasets using scMustree. **(a)** UMAP projection of single-cell transcriptomes, with coloring based on different branches of the scMustree hierarchy. **(b)** The complete hierarchy of DCs constructed by scMustree, with node colors denoting major branches. **(c)** Cross-dataset validation showing the similarity of marker expression patterns between established DC subtypes and corresponding scMustree-identified branches. **(d)** UMAP projection of single-cell transcriptomes, color-coded by BC subtypes. **(e)** The complete hierarchy of BCs constructed by scMustree, with node colors denoting dominant cell subtype annotations. **(f)** Heatmap visualization of differentially expressed genes across scMustree-identified sub-branches.

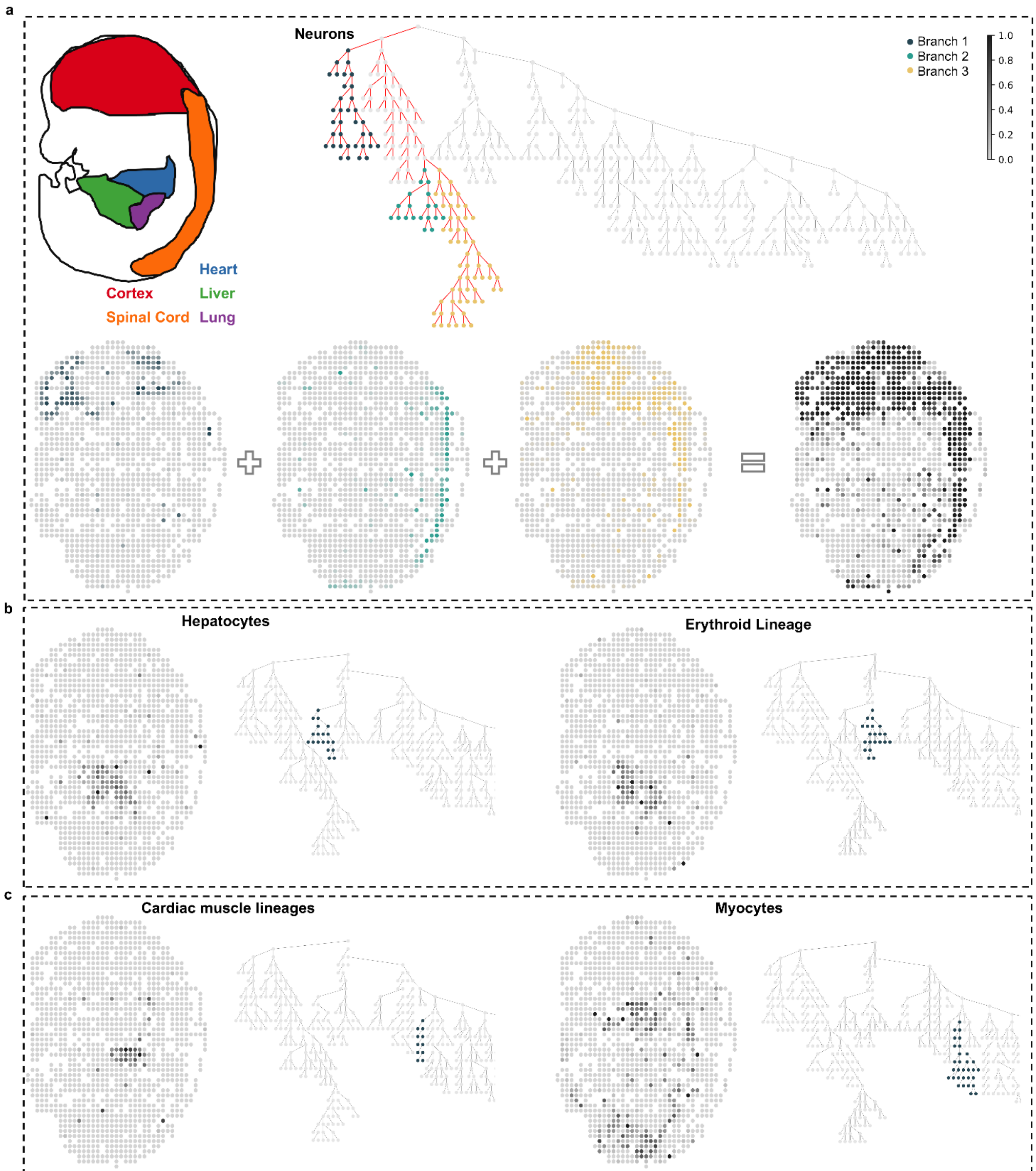

Figure S12. Analysis of spatial distribution and functional associations of cell types revealed by scMustree. **(a)** Anatomical regions of Slide 14 in mouse embryo sagittal sections (left) and the hierarchy generated by scMustree (right). The neuron-dominated sub-hierarchy is highlighted with red edges, and three main branches are color-coded. The spatial distribution of cells in the corresponding branches and sub-hierarchies is shown at the bottom. Spots are marked with a continuous gradient of the same color to indicate the proportion of cells from the same branches or sub-hierarchy at each spatial coordinate. **(b)** Spatial

distribution patterns of cells within branches dominated by Hepatocytes and Erythroid Lineage cells. **(c)** Spatial distribution patterns of cells within branches dominated by cardiac muscle lineages and myocytes.

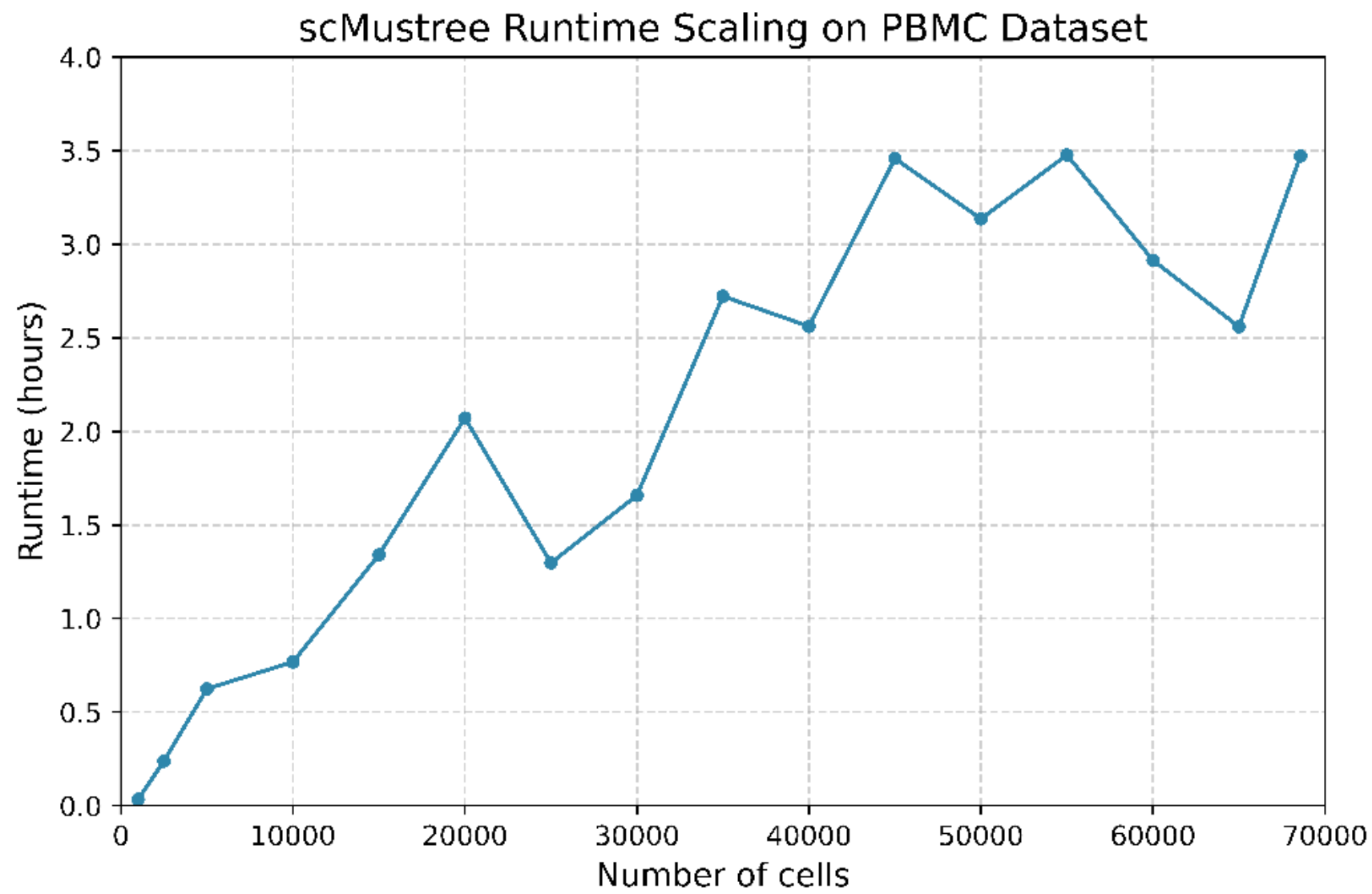

Figure S13. Runtime scaling of scMustree with increasing cell number. Runtime (in hours) was recorded for subsampled datasets derived from a PBMC dataset of 68,579 cells, with cell numbers ranging from 1,000 to 68,579.

Supplementary Tables

Table S1. Detailed information on the datasets used in this study.

Dataset	Cells	Genes	Types	Accession	Description
Airway	7193	27716	7	GSE103354	mouse tracheal epithelium cells
Darmanis	466	22085	9	GSE67835	human brain
Hippocampus	1188	25392	8	GSE84371	mouse hippocampus
iLNs	9662	25929	24	GSE139600	murine iLNs
Koh	498	60483	9	GSM2257302	human embryonic stem cells
Li	561	57241	7	GSE81861	human colorectal tumors
Macosko	14653	11422	39	GSE63473	mouse retina cells
MacParland	8444	5000	11	GSE115469	human liver cells
Mammary	13684	27998	13	GSE150580	murine mammary tissues
Pancreas	8569	20125	14	GSE84133	human pancreas
Puram	3363	23686	8	GSE103322	non-malignant cells in Head and Neck Cancer
Glaucoma	18203	33660	11	SCP780	cells from human TM and surrounding tissues
Alzheimer1	118240	60706	13	GSE212606	human brain cells from the hippocampus and the superior and middle temporal gyrus (SMTG) regions
Alzheime2	64020	29902	/	GSE254205	human cells from fresh-frozen frontal cortex tissue of individuals diagnosed with AD who carried either the APOE4/4 or the APOE3/3 genotype.
Dendritic cells	2099	32738	1	10x Genomics	dendritic cells in human peripheral blood mononuclear cells
Villani et al.	1078	26593	/	SCP43	human blood dendritic cells, monocytes, and progenitors
Bipolar cells	1276	6198	1	GSE201402	bipolar cells in mouse retina
sci-Space	17309	52636	20	GSE166692	sagittal sections of whole E14 mouse embryos

Table S2. Normalized mutual information (NMI) of Louvain clustering across different resolution parameters, with the selected resolution indicated for each dataset.

	0.1	0.2	0.3	0.4	0.5	0.6	0.7	0.8	0.9	1	Selected Resolution Parameter
Airway	0.3789	0.4135	0.4081	0.4088	0.4280	0.4169	0.5003	**0.5073**	0.5004	0.5140	0.8
Hippocampus	0.5447	0.5436	0.6098	0.5877	0.5769	0.5989	0.6092	0.5761	**0.5790**	0.5933	0.9
iLNs	0.6312	0.6729	0.6791	0.6970	0.7715	0.7710	0.7623	0.7675	0.7673	**0.7706**	1.0
Mammary	0.5437	0.5820	0.5918	0.5929	0.7331	0.7259	0.7077	0.7387	**0.7464**	0.7370	0.9
Pancrea	0.5975	0.6490	0.7677	0.7578	0.7721	0.7715	0.7791	0.7596	**0.7915**	0.7841	0.9
Darmanis	0.6179	0.7171	0.7165	0.7194	0.7186	0.7217	0.7214	0.7201	0.7439	**0.7502**	1.0
Koh	0.8295	**0.9655**	**0.9655**	**0.9655**	**0.9655**	0.9458	0.9458	0.9458	0.9458	0.9458	0.2~0.5
Li	0.6674	0.8129	0.8414	0.8444	0.8444	**0.8914**	**0.8914**	**0.8914**	**0.8914**	**0.8914**	0.6~1.0
Puram	0.5696	0.5943	0.5949	0.6037	0.6038	0.6037	**0.7088**	0.6846	0.7001	0.6779	0.7
Macosko	0.6873	0.7989	0.8177	0.8352	**0.8279**	0.8259	0.8153	0.8287	0.8296	0.8330	0.5
MacParland	0.5775	0.5854	0.5850	0.7162	0.7123	0.7077	0.7055	0.7045	0.7105	**0.7462**	1.0

Table S3. Adjusted Rand index (ARI) of Louvain clustering across different resolution parameters, with the selected resolution indicated for each dataset.

	0.1	0.2	0.3	0.4	0.5	0.6	0.7	0.8	0.9	1	Selected Resolution Parameter
Airway	0.0972	0.1376	0.1381	0.1383	0.1565	0.1379	0.2706	**0.2836**	0.2550	0.2771	0.8
Hippocampus	0.3271	0.3243	0.4783	0.4209	0.3897	0.3760	0.3848	0.4897	**0.4921**	0.3613	0.9
iLNs	0.2894	0.4318	0.4828	0.5159	0.7073	0.7095	0.6832	0.7117	0.7049	**0.7135**	1.0
Mammary	0.1164	0.1670	0.2011	0.1916	0.4906	0.4755	0.4154	0.5019	**0.5090**	0.4964	0.9
Pancrea	0.1813	0.2770	0.5001	0.4886	0.5043	0.5042	0.5227	0.4853	**0.5394**	0.5243	0.9
Darmanis	0.3924	0.6207	0.6336	0.6419	0.6459	0.6430	0.6418	0.6432	0.7402	**0.7425**	1.0
Koh	0.7498	**0.9646**	**0.9646**	**0.9646**	**0.9646**	0.9177	0.9177	0.9177	0.9177	0.9177	0.2~0.5
Li	0.3736	0.6427	0.6939	0.6975	0.6975	**0.7939**	**0.7939**	**0.7939**	**0.7939**	**0.7939**	0.6~1.0
Puram	0.1843	0.2140	0.2162	0.2341	0.2344	0.2340	**0.4282**	0.3692	0.4132	0.3618	0.7
Macosko	0.4975	0.8188	0.8659	0.8680	**0.8722**	0.8560	0.8297	0.8499	0.8494	0.8531	0.5
MacParland	0.1165	0.1292	0.1362	0.3838	0.3699	0.3430	0.3295	0.3232	0.3272	**0.4182**	1.0

Table S4. Cell-type-specific marker genes for expression analysis in the Puram dataset.

Cell Type	Markers
T cells	CD3D, CD3E, CD8A, CD4, IL7R
B/plasma cells	CD19, MS4A1, CD79A, CD79B
Macrophages	CD68, CD14, CD163, FCGR3A, MRC1, C1QA
Dendritic cells	CD1C, CLEC10A, FCER1A, CD83, LILRA4, CLEC4C, IRF7, TLR7, CLEC9A, XCR1, BATF3
Mast cells	TPSB2, CPA3, KIT, FCER1A
Endothelial cells	PECAM1, VWF, CDH5, CLDN5, ENG, FLT1
Fibroblasts	COL1A1, COL3A1, DCN, LUM, PDGFRA, FAP, THY1, ACTA2
Myocytes	ACTA2, MYH11, TAGLN, TNNT2, MYH6, MYH7, TTN, ACTN2

Table S5. Performance comparison (NMI) of scMustree and its three ablation variants across all 11 benchmark datasets.

	scMustree	scMustree-single	scMustree-Euclidean	scMustree-Kendall
Airway	0.637	**0.638**	0.295	0.310
Hippocampus	**0.881**	0.813	0.498	0.512
iLNs	0.718	**0.737**	0.613	0.544
Mammary	0.837	**0.880**	0.136	0.603
Pancrea	0.856	**0.912**	0.543	0.651
Darmanis	**0.773**	0.667	0.625	0.686
Koh	**0.953**	0.942	0.751	0.809
Li	0.925	**0.953**	0.664	0.760
Puram	0.839	**0.844**	0.523	0.615
Macosko	**0.833**	0.648	0.706	0.706
MacParland	**0.899**	0.851	0.584	0.731
Mean	**0.832**	0.808	0.540	0.630

Table S6. Performance comparison (ARI) of scMustree and its three ablation variants across all 11 benchmark datasets.

	scMustree	scMustree-single	scMustree-Euclidean	scMustree-Kendall
Airway	**0.697**	0.692	0.103	0.273
Hippocampus	**0.935**	0.671	0.080	0.121
iLNs	0.693	**0.725**	0.353	0.161
Mammary	0.879	**0.918**	0.104	0.338
Pancrea	0.808	**0.941**	0.116	0.505
Darmanis	**0.720**	0.640	0.318	0.493
Koh	**0.944**	0.933	0.417	0.628
Li	0.949	**0.962**	0.343	0.683
Puram	0.713	**0.722**	0.119	0.578
Macosko	**0.883**	0.713	0.393	0.403
MacParland	**0.951**	0.736	0.179	0.846
Mean	**0.834**	0.787	0.230	0.457

Table S7. DC subtypes -specific marker genes used for Cross-dataset validation.

Cell Type	Markers
DC1	CLEC9A, HLA-DPA1, CADM1, CAMK2D, CPVL, WDFY4, CPNE3, IDO1, HLA-DPB1, HLA-DOB, HLA-DQB1, CLNK, CSRP1, SNX3, ZNF366, KIAA1598
DC2	CD1C, FCER1A, CLEC10A, ADAM8, CD1D, FCGR2B, CLEC4A, SLC2A3, CD33, ETS2, CLIC2, PEA15, CACNA2D3, CD1E, MBOAT7, NR4A2, AGPAT9, ENTPD1, CD2
DC3	S100A9, S100A8, VCAN, LYZ, ANXA1, PLBD1, RNASE2, CD163, CSF3R, MNDA, CD14, NAIP, CSTA, FCN1, FPR1, F13A1
DC4	FCGR3A, FTL, SERPINA1, LST1, AIF1, SAT1, CTSS, MTSS1, TCF7L2, PSAP, FTH1, IFITM3, MS4A7, LILRB2, PILRA, CSF1R, ASAH1, LRRC25, HLA-E
DC5	AXL, PPP1R14A, SIGLEC6, CD22, DAB2, S100A10, FAM105A, ALDH2, LTK, DPYSL2, LGMN, IRF4, PLAC8, CCND3, MYO1E, SLC41A2, SCN9A, SIGLEC1
DC6	GZMB, IGJ, SERPINF1, ITM2C, PLD4, CCDC50, IRF7, PTPRS, ALOX5AP, TCF4, BCL11A, LILRA4, FAM129C, MZB1, UGCG, DERL3

Supplementary Notes

Supplementary Note 1 Tree-Pruning Algorithm for Hierarchical Clustering Evaluation.

This note describes the bottom-up tree-pruning algorithm used to extract a flat clustering from a given hierarchical tree (dendrogram) for subsequent evaluation against reference cell-type annotations. The algorithm searches for a set of non-overlapping nodes whose combined cell assignment optimises a heuristic score that balances node purity and global recall, while discouraging over-fragmentation.

Node scoring function (class-aware).

To prevent rare cell types from being overshadowed by large populations, we define a score for each node v and cell type c as the product of cluster purity and global recall:

$$\mathrm{Score}_c(v) = \mathrm{Purity}_c(v) \times \mathrm{Recall}_c(v)$$

where

$$\mathrm{Purity}_c(v) = \frac{n_{v,c}}{n_v}$$

$$\mathrm{Recall}_c(v) = \frac{n_{v,c}}{N_c}$$

with $n_{v,c}$ being the number of cells of type c in node v, n_v the total cell count in node v, and N_c the total number of cells of type c in the entire dataset. The final score of a node is taken as the maximum across all cell types:

$$\mathrm{Score}(v) = \max(\mathrm{Score}_c(v))$$

Tree dynamic programming (DP).

The pruning problem is decomposed into independent "keep vs. split" decisions on the binary tree. Let $\mathrm{DP}(v)$ denote the maximum heuristic score obtainable from the subtree rooted at node v (without any cluster-count penalty). The recurrence is:

$$\mathrm{DP}(v) = \max(\mathrm{Score}(v), \mathrm{DP}(v.\mathrm{left}) + \mathrm{DP}(v.\mathrm{right}))$$

For leaf nodes, $\mathrm{DP}(v) = \mathrm{Score}(v)$. This recurrence is evaluated via a bottom-up post-order traversal.

Top-K extension.

Because the base DP keeps only the single best solution per node, which may discard combinations that are suboptimal locally but globally preferable (since ARI is not additive), each node stores up to $K = 50$ candidate solutions with the highest scores. Each candidate is a tuple (score, nodes_list, cluster_count).

For an internal node v, the candidate pool is built from:

- *Keep*: the solution consisting of node v alone, with score $\mathrm{Score}(v)$ and cluster count 1.
- *Split*: all pairwise combinations of one candidate from the left subtree and one from the right subtree; the combined score is the sum of the two scores, and the cluster count is the sum of the two counts.

The pool is sorted by score descending, duplicates (identical node sets) are removed, and the top K candidates are retained and passed to the parent.

Regularisation penalty.

To penalise overly fragmented solutions, a penalty proportional to the number of clusters is subtracted from the score of each candidate:

$$\mathrm{FinalScore}(p) = \sum_{v \in p} \mathrm{Score}(\mathrm{v}) - \alpha \times C_p$$

where $\alpha = 0.05$ is the regularisation coefficient and C_p is the number of clusters in candidate solution p. This penalised score is used for sorting and truncation at every internal node.

Final selection at the root.

After processing the root, the top K candidates are obtained. For each candidate (a set of non-overlapping nodes), we construct a predicted label vector of length N (number of cells) by assigning a unique cluster ID to every selected node and propagating that ID to all cells within it. The candidate that achieves the highest ARI against the ground-truth labels is chosen as the final clustering output; its corresponding NMI is also recorded.

Supplementary Note 2 Usage of comparative methods.

To comprehensively assess the performance of cellular tree construction methods, we conduct a benchmark analysis comparing scMustree with other methods.

To construct the multi-resolution tree structure using the Louvain algorithm, we first apply the standard normalization workflow from the *Scanpy* Python package (version 1.10.2). We then use the *best_partition* function from the *community* package with *resolution* parameters ranging from 0.1 to 1.0 to obtain clustering results at various resolutions. Based on the overlap of clusters between adjacent resolution levels, we draw an edge between two clusters if their overlap exceeds 30%, thereby capturing the associations between cells at different resolutions. The scSHC package is obtained from GitHub (igrabski/sc-SHC). Using its internal functions with default parameters, we first obtain a cell distance matrix as the Euclidean distance on the latent variables estimated by the approximate generalized linear model principal components analysis (GLM-PCA) procedure using 2,500 genes. We then apply hierarchical clustering with Ward's criterion to construct the tree structure. The SEAT package is obtained from GitHub (deepomicslab/SEAT). Specifically, the UMAP embedding is first obtained using the standard normalization workflow from the Scanpy Python package, and then the default parameters of SEAT are used to generate results from which the 'Newick' tree structure is extracted. The scDCC and scGNN packages are obtained from GitHub (ttgump/scDCC and juexinwang/scGNN, respectively). Both methods use clustering results obtained with default parameters, except that for scDCC we also provided the true number of cell types as input (instead of inferring it) and, for some smaller datasets, reduced the '*pretrain*_epochs' parameter to avoid errors.

Supplementary Note 3 Identification of differential genes.

A traditional Wilcoxon rank-sum test was used to identify differentially expressed (DE) genes with an FDR cutoff of 0.05 and an inter-group absolute fold-change cutoff of 1.5. Fold-change values were measured based on the group-wise mean expression values of each gene. A gene was considered cluster-specific if it was found to be differentially up-regulated in a particular cluster compared to each of the remaining clusters.

Supplementary Note 4 Gene Ontology functional enrichment analysis.

GO functional enrichment analysis was performed using the enrichr function from the gseapy Python package (version 1.1.4). The gene sets used included 'GO_Biological_Process_2023', 'GO_Cellular_Component_2023', and 'GO_Molecular_Function_2023'.

Supplementary Note 5 scMustree yields high-purity terminal populations

Accurately determining the terminal populations is critical for constructing a reliable hierarchy. To evaluate the accuracy of the decomposed clusters (as terminal populations) obtained by scMustree, we conduct a quantitative analysis of the accuracy of clusters before and after decomposition. For each cluster, we first calculate its purity based on the annotation information, defined as the proportion of the most abundant cell type within the cluster. The purity of cluster i is calculated as follows: $\text{Purity}_i = \frac{\max(N_i^1, N_i^2, \ldots, N_i^T)}{N_i}$, where N_i is the total number of cells in cluster i, N_i^j is the number of cells of type j in cluster i, and T is the total number of cell types in the dataset. We compute the average cluster purity distribution before and after decomposition for all datasets (Fig. S6a). The average purity for each dataset is shown in Fig. S6b. The results demonstrate that most terminal populations are predominantly composed of a single cell type.

For datasets containing rare cell types (<1% abundance), we develop a rare-type coverage metric. First, we identify the most abundant cell type in each cluster, remove duplicates, and obtain the set of types before and after decomposition, denoted as C. The set of rare cell types in the data is defined as C_{rare}. The coverage of rare cell types is calculated as $\text{Coverage}_{\text{rare}} = \left(\frac{|C \cap C_{\text{rare}}|}{|C_{\text{rare}}|}\right) \times 100\%$. Fig. S6c reveals that decomposed clusters achieve substantially better rare-type coverage than initial clustering, with previously undetected rare types in some datasets being effectively identified.

Additionally, we examine the proportion of cells involved in clusters primarily composed of rare cell types. This proportion is calculated as follows: $\text{Proportion} = \left(\frac{\sum_{r \in C_{rare}} \frac{N_r'}{N_r}}{|C_{\text{rare}}|}\right) \times 100\%$, where N_r' is the total number of cells of rare type r in clusters primarily composed of type r, and N_{r} is the total number of cells of rare type r. The average proportion of cells involved in rare cell types for each dataset before and after decomposition is visualized in Fig. S6d. The results indicate that most major rare cell types (with a proportion greater than 60%) are effectively separated after decomposition. In summary, scMustree achieves high-purity terminal populations by iteratively decomposing initial clusters through hierarchical clustering.

Supplementary Note 6 Ablation Analysis of scMustree Components.

To dissect the contributions of scMustree's core algorithmic components, we perform a systematic ablation study across all 11 benchmark datasets. We design three variant configurations, each disabling or replacing one major component while keeping the rest of the pipeline unchanged:

(1) scMustree-single: This variant replaces the iterative cluster decomposition procedure with a single round of high-resolution Louvain clustering. The resolution parameter was tuned so that the number of resulting leaf clusters was as close as possible to that produced by the full scMustree pipeline. This configuration is designed to evaluate the contribution of the iterative decomposition strategy.

(2) scMustree-Euclidean: This variant retains the iterative cluster decomposition procedure but replaces the CFR-based RBO similarity with Euclidean distance between cluster-level pseudobulk expression profiles (average expression vector of each cluster). This configuration is designed to evaluate the contribution of the CFR-based functional ranking and RBO similarity measure relative to a conventional distance-based similarity metric.

(3) scMustree-Kendall: This variant retains both the iterative cluster decomposition and the cluster-specific functional ranking (CFR), but replaces Rank-Biased Overlap (RBO) with

Kendall's tau, which measures global concordance between two full rankings rather than emphasizing top-ranked agreement. This configuration is designed to evaluate whether the top-weighted emphasis of RBO provides additional value over a global ranking correlation metric.

We evaluate all three variants using the same oracle pruning protocol and performance metrics (NMI and ARI) as described in Supplementary Note 1. The complete results are reported in Tables S5 (NMI) and S6 (ARI).

The ablation results provide several insights into the contribution of each component.

First, the CFR-based RBO similarity measure is the most influential component. Replacing it with Euclidean distance between pseudobulk expression profiles caused the most substantial performance drop, with mean NMI decreasing from 0.832 to 0.540 and mean ARI from 0.834 to 0.230. This suggests that functional ranking-based similarity captures biologically meaningful relationships between clusters far more effectively than conventional expression-distance-based approaches.

Second, the CFR representation itself contributes substantially to performance. Compared with scMustree-Euclidean, scMustree-Kendall—which retains CFR but replaces RBO with Kendall's tau—achieved notably higher average performance (mean NMI: 0.630 vs. 0.540; mean ARI: 0.457 vs. 0.230). This indicates that representing inter-cluster relationships as cluster-specific functional rankings provides useful biological information beyond simple expression proximity. However, the full scMustree model still clearly outperformed scMustree-Kendall, demonstrating that the top-weighted comparison provided by RBO is essential for effectively leveraging CFRs.

Third, the iterative decomposition strategy improves average performance and robustness, particularly for datasets with complex cellular heterogeneity. We acknowledge an important caveat in the design of the scMustree-single variant: to ensure a fair comparison of the clustering module, we intentionally tune its resolution to match the number of leaf clusters produced by the full iterative model. Effectively, this means we instruct the single-round clustering to operate at an externally defined, optimal granularity—a target that the full scMustree model must discover entirely autonomously through its iterative process. This experimental setup, while necessary for controlled comparison, inherently introduces a bias that partially underestimates the adaptive value of the iterative decomposition strategy, as it provides the single-round baseline with privileged knowledge of the ideal terminal population count. With this potential limitation in mind, the scMustree-single variant performs comparably to, or slightly better than, the full model on some datasets with relatively well-separated cell populations, such as Pancrea, Mammary, and Li. However, the full scMustree model still achieves higher average NMI and ARI across all datasets and shows markedly stronger performance on more complex datasets such as Hippocampus, Macosko, and MacParland. These results suggest that while a single high-resolution clustering step may be sufficient for relatively simple datasets, the iterative decomposition strategy is substantially more beneficial for resolving deeper or more heterogeneous hierarchical structures, especially given that the full model achieves this without any prior knowledge of the optimal cluster count.

Fourth, the comparison between scMustree-Kendall and the full model highlights the value of RBO's top-weighted design. Kendall's tau evaluates agreement across the entire ranking, whereas RBO emphasizes agreement among the top-ranked clusters. Since the top-ranked clusters represent the closest functional neighbors of a reference cluster, emphasizing top-ranked overlap is better aligned with the biological goal of identifying local functional similarity.

In summary, the ablation analysis confirms that the performance gains of scMustree arise from the synergistic combination of its core components: the CFR-based similarity measure

provides the largest improvement over conventional approaches; RBO further enhances the use of CFRs by prioritizing top-ranked functional neighbors; and iterative decomposition improves robustness and enables resolution of complex hierarchical structures.

Supplementary Note 7 scMustree infers cell-type relationships validated across multiple species

Understanding cellular interactions within the aqueous humor outflow pathways provides critical insights into glaucoma pathogenesis and therapeutic development[1,2]. Previous single-cell RNA sequencing studies have established a foundational atlas of adult trabecular meshwork (TM) and adjacent tissues, identifying 19 histologically validated cell types[3]. This framework enables systematic cross-species comparisons in cynomolgus macaque, rhesus macaque, porcine, and murine models. Building upon this foundation, Mah et al. develop an evolutionary model for phylogenetic cell-type classification using multi-species datasets[4]. To facilitate a direct comparison with Mah et al.'s cross-species cell phylogeny structure, we focus on 11 consensus cell types (18,203 cells) retained in their evolutionary framework. The spatial relationships among these types are visualized through UMAP dimensionality reduction (Fig. S7a).

scMustree reconstructs a multi-scale hierarchy that fully captures the relationships among the 11 cell types (Fig. S7b). We further refine the topology to compare key branching patterns (Fig. S7c), thereby showcasing scMustree's capacity to identify biologically meaningful proximities that align with cross-species phylogenetic frameworks. Specifically, immune cell types (macrophages and NKT cells) form adjacent branches reflecting shared immune surveillance functions[5]. Schwann cell subtypes (myelinating and non-myelinating) are grouped according to developmental lineage[6]. Meanwhile, the ‘vessel endothelia’ types (endothelia, Schlemm's canal, collector channel cells) share canonical endothelial markers, following the classification by van Zyl et al[3].

A notable divergence emerges in pericyte positioning. Mah et al. associate pericytes with the ciliary muscle, but scMustree positions them adjacent to vascular endothelia. This topological realignment more accurately reflects in vivo biology, as pericytes positioned adjacent to vascular endothelia regulate capillary dynamics and blood flow, aligning with their well-established role in vascular homeostasis[7]. In contrast, the ciliary muscle primarily contributes to tissue structure and contractile function essential for lens accommodation[8], making its association with pericytes less consistent with their known physiological function.

We integrate the above functionally related cell types to reduce redundancy among functionally overlapping types, allowing for clearer observation of inter-type distinctions in topological comparisons (Fig. S7d). The hierarchical merging in scMustree starts with the integration of pericytes and vascular endothelia, which is anatomically justified by their shared basement membrane localization. It then progressively incorporates juxtacanalicular tissue, Schwann cells, melanocytes, immune clusters, and ciliary muscle. This stepwise integration pattern recapitulates the spatial gradient from the vascular core (pericytes-endothelia) to adjacent outflow structures (JCT), ultimately reaching distal contractile elements (ciliary muscle).

Detailed analysis of non-myelinating Schwann cells (SchwannCell-nmy) reveals three functionally distinct subclusters (Fig. S7e). Differential expression analysis (Supplementary Data 4) identifies branch-specific markers, coupled with Gene Ontology (GO) enrichment (Fig. S7f~h) confirming functional divergence. Branch 1 demonstrates stress-response specialization, highlighted by molecular chaperones *CRYAB* and *HSPB1*, interferon regulators *IFITM3* and *ISG15*, and chemokine signaling through *CCL2*. GO terms "Response to Type II Interferon" and

"Ribosomal subunit" reflected inflammation-adaptation duality. Branch 2 exhibits epithelial-ionic regulation with *KRT13* and *KRT19* for barrier maintenance, *CA2/CA4* mediating "Carbon dioxide transport", and *AQP1/3* maintaining osmotic balance. Stress-adaptation genes *HSPA1B* and *ZFP36L1* suggest microenvironmental modulation. Branch 3 specializes in immune coordination, dominated by antigen presentation genes *HLA-DRA* and *CD163*, inflammatory regulators *CEBPD* and *TSC22D3*, alongside translation machinery *RPL/RPS* family. Enriched "MHC class II antigen presentation" and "Regulation of cytokine production" confirm adaptive immune crosstalk.

These findings demonstrate scMustree's multi-scale capability to validate evolutionarily cell-type relationships while resolving functional diversity within cellular types. The hierarchical architecture preserves the global topological features of TM ecosystems while enabling granular analysis of subtype-specific pathophysiology, providing a powerful framework for linking functional diversity to disease mechanisms.

Supplementary Note 8 scMustree deciphers patient-specific cell subtypes in Alzheimer's disease via phenotype-driven topological disparities

To assess the utility of scMustree in deciphering patient-specific gene expression heterogeneity and disease mechanisms, we analyzed the Alzheimer's disease (AD) single-cell dataset from Haney et al. [9]. The dataset contains ~64,000 cells from 10 APOE 4/4 genotype and 8 APOE 3/3 genotype patients. While UMAP visualizations show batch effects across individuals, scMustree's hierarchies reduce these technical biases through its node-merging algorithm based on characteristic gene expression (Figs. S8a and S8b). Hierarchies built for each genotype independently exhibit consistent topology (Figs. S8c and S8e), with high agreement in cell type annotation and cellular composition (Figs. S8d, S8f, and S8g), confirming method robustness. Notably, when comparing neuron-dominant branches (Branch 4 vs. 5), APOE 4/4 cells display disease-associated neuronal phenotypes (DANs)—characterized by elevated expression of heat shock protein, calmodulin, and ubiquitin B genes (consistent with PS19-APOE4 mouse hippocampal pathology). These neuronal APOE4-promoted DANs (nE4-DANs) show APOE4-specific overexpression and correlate with tau pathology and hippocampal atrophy[10].

At the individual level, scMustree identifies an anomalous sample (0406) in the APOE 4/4 cohort (Fig. S9). This specimen exhibits distinct hierarchical topology, with oligodendrocyte marker analysis revealing predominant oligodendrocyte composition, suggesting sample processing artifacts. This demonstrates scMustree's utility for sample quality control. When comparing oligodendrocyte subtypes across patients 0354, 0527, and 1369 (Fig. S10a), the first two show a dual-branch pattern, while the latter had a single-branch architecture. Differential gene analysis and Jaccard similarity (Fig. S10b) reveal high homology (~50%) in the "emerging subtype" between dual-subtype patients, with the "conserved subtype" maintaining ~50% similarity across all three. Clinical-pathological records indicate that 0354 and 0527 exhibit cerebral white-matter rarefaction (CWMR). Functional enrichment of the emerging subtype implicate core pathological mechanisms: activation of myelination ($P=5.97\times10^{-13}$), cholesterol biosynthesis ($P=1.0 \times 10^{-5}$), sphingolipid metabolism ($P=6.92\times10^{-6}$), and oligodendrocyte differentiation ($P=1.53\times10^{-4}$)—processes directly corresponding to AD white matter pathology features (demyelination, lipid dysregulation, glial dysfunction). Collectively, scMustree interprets patient-specific cellular subtypes through topological disparities, advancing pathological subtyping and revealing potential therapeutic targets.

Supplementary Note 9 scMustree effectively characterizes dendritic and bipolar cell subtypes through hierarchy construction

In cellular biology research, the precise identification and characterization of cellular subtypes have become paramount for understanding functional diversity. This is particularly crucial for dendritic cells (DCs) and bipolar cells (BCs), where subtype variations determine their distinct roles in immune responses[11] and neural signaling[12], respectively. Building upon previous subtype characterization efforts, we demonstrate how scMustree's hierarchical architecture effectively resolves cellular subtypes in these critical cell subtypes.

Using 2,099 DCs extracted from the 68k PBMC dataset, we evaluate scMustree's ability to identify subtypes beyond original annotations. UMAP visualization reveals spatial relationships among DCs (Fig. S11a), while scMustree generates a multi-scale hierarchy with five principal branches (Fig. S11b). To validate these branches, we perform cross-dataset correlation analysis comparing the transcriptional profiles of cells within each branch against Villani et al.'s FACS-validated DC subtypes[13] using their established marker set (Fig. S11c, marker list in Table S7). The analysis reveals distinct subtype associations: Branches 1 and 2 exhibit the strongest correlations with DC1 (r=0.778) and DC2 (r=0.720), respectively, while Branches 3 and 4 both show high similarity to DC4 (r=0.836 and 0.754). Notably, Branch 5 demonstrates significant alignment with plasmacytoid DCs (pDCs/DC6, r=0.777). The observed transcriptional gradients between adjacent branches suggest a topological hierarchy in subtype relationships, potentially reflecting developmental or functional continuities within the DC subtype.

Analysis of 6,198 BCs from the mouse retina dataset, which were originally annotated as ten subtypes[14], reveals scMustree's capacity to not only recapitulate known subtypes but also identify novel subdivisions. UMAP visualization reveals spatial relationships among BCs (Fig. S11d), while scMustree generates a multi-scale hierarchy with clear branch correspondence to annotated subtypes (Fig. S11e). Additionally, we observe heterogeneous subtypes, like BC2 splitting into three branches and BC10 into two. Differential gene expression analysis is done on each branch (Fig. S11f). Results show that though branches of the same subtype share certain gene expression patterns, they also have remarkable gene expression differences, suggesting they may have different functional roles.

These results position scMustree as a powerful tool for multi-scale subtype discovery, capable of both validating established classifications and revealing novel cellular subdivisions through its hierarchical analytical framework.

Supplementary Note 10 scMustree reveals spatial distribution relationships of cell types

The spatial distribution patterns of cell types are closely associated with their physiological functions. To validate scMustree's capability in deciphering spatial distribution relationships between cell types, we apply it to the sci-Space dataset[15] containing 14 sagittal sections from two E14.0 mouse embryos, which provides spatial coordinates and whole transcriptome data for approximately 17,000 nuclei. scMustree constructs a hierarchy based solely on RNA modality data without spatial coordinate reference. Using the mouse embryo anatomical regions as a reference, we analyze the spatial distribution of cells within hierarchy nodes (Fig. S12a).

Specifically, we quantify the proportion of cells in each spatial coordinate within the nodes of its sub-hierarchies and visualize their spatial distribution using a continuous color gradient. Our analysis reveals a sub-hierarchy in which nodes are predominantly distributed in the Cortex and Spinal Cord, and these nodes are composed of neurons. Further examination of the spatial distribution of cells within the main branches of this sub-hierarchy (Fig. S12a(bottom)) shows

significant spatial associations between neighboring branches. Similarly, we observe another sub-hierarchy where cells within nodes are primarily distributed in the liver (Fig. S12b). This sub-hierarchy contains two branches composed mainly of Hepatocytes and Erythroid Lineage cells, reflecting their spatial proximity. During mouse embryo development, these cells are predominantly located in the fetal liver.

Additionally, we investigate cardiac muscle lineages and myocytes, both derived from mesodermal myogenic progenitors during embryonic development. These cell types share key transcription factors and structural proteins. Although they do not exhibit significant spatial associations, their functional relationship is reflected in the hierarchy as location proximity (Fig. S12c). In summary, scMustree not only reveals spatial distribution relationships among cell types but also maintains functional associations between them.

Supplementary Note 11 Scalability analysis of scMustree

To evaluate the computational scalability of scMustree, we performed a systematic runtime analysis on a large-scale PBMC dataset containing 68,579 cells. Subsampled datasets of varying sizes (ranging from 1,000 to 68,579 cells) were generated, and the runtime of scMustree was recorded for each subset. As shown in Figure S13, the runtime does not increase proportionally with the total number of cells. Instead, the computational complexity is primarily determined by the number of terminal populations (decomposed clusters) generated during the iterative decomposition process, which remains relatively stable once sufficient cellular diversity is captured. This property enables scMustree to maintain efficient performance even when applied to datasets exceeding one hundred thousand cells, supporting its applicability to large-scale single-cell studies.

References

1. Wang, N., Chintala, S. K., Fini, M. E. & Schuman, J. S. Activation of a tissue-specific stress response in the aqueous outflow pathway of the eye defines the glaucoma disease phenotype. *Nat Med* **7**, 304–309 (2001).
2. Thomson, B. R. *et al.* Cellular crosstalk regulates the aqueous humor outflow pathway and provides new targets for glaucoma therapies. *Nat Commun* **12**, 6072 (2021).
3. Van Zyl, T. *et al.* Cell atlas of aqueous humor outflow pathways in eyes of humans and four model species provides insight into glaucoma pathogenesis. *Proc. Natl. Acad. Sci. U.S.A.* **117**, 10339–10349 (2020).
4. Mah, J. L. & Dunn, C. W. Cell type evolution reconstruction across species through cell phylogenies of single-cell RNA sequencing data. *Nat Ecol Evol* **8**, 325–338 (2024).
5. Perez, V. L. & Caspi, R. R. Immune mechanisms in inflammatory and degenerative eye disease. *Trends in Immunology* **36**, 354–363 (2015).
6. Jessen, K. R. & Mirsky, R. The origin and development of glial cells in peripheral nerves. *Nat Rev Neurosci* **6**, 671–682 (2005).
7. Alarcon-Martinez, L. *et al.* Pericyte dysfunction and loss of interpericyte tunneling nanotubes promote neurovascular deficits in glaucoma. *Proc. Natl. Acad. Sci. U.S.A.* **119**, e2110329119 (2022).
8. Wiederholt, M., Thieme, H. & Stumpff, F. The regulation of trabecular meshwork and ciliary muscle contractility. *Progress in Retinal and Eye Research* **19**, 271–295 (2000).
9. Haney, M. S. *et al.* APOE4/4 is linked to damaging lipid droplets in Alzheimer's disease microglia. *Nature* **628**, 154–161 (2024).
10. Blumenfeld, J., Yip, O., Kim, M. J. & Huang, Y. Cell type-specific roles of APOE4 in Alzheimer disease. *Nat. Rev. Neurosci.* **25**, 91–110 (2024).
11. Shortman, K. & Liu, Y.-J. Mouse and human dendritic cell subtypes. *Nat Rev Immunol* **2**, 151–161 (2002).
12. Shekhar, K. *et al.* Comprehensive Classification of Retinal Bipolar Neurons by Single-Cell Transcriptomics. *Cell* **166**, 1308-1323.e30 (2016).
13. Villani, A.-C. *et al.* Single-cell RNA-seq reveals new types of human blood dendritic cells, monocytes, and progenitors. *Science* **356**, eaah4573 (2017).
14. Dou, J. *et al.* Bi-order multimodal integration of single-cell data. *Genome Biol* **23**, 112 (2022).
15. Srivatsan, S. R. *et al.* Embryo-scale, single-cell spatial transcriptomics. *Science* **373**, 111–117 (2021).
